# Multiresidues Multiclass Analytical Methods for Determination of Antibiotics in Animal Origin Food: A Critical Analysis

**DOI:** 10.3390/antibiotics12020202

**Published:** 2023-01-18

**Authors:** Sílvia Cruz Barros, Ana Sanches Silva, Duarte Torres

**Affiliations:** 1 National Institute for Agricultural and Veterinary Research (INIAV), I.P, 4485-655 Vila do Conde, Portugal; 2 EPIUnit–Institute of Public Health, University of Porto, 4200-450 Porto, Portugal; 3 Faculty of Nutrition and Food Sciences, University of Porto, 4200-393 Porto, Portugal; 4 Laboratory for Integrative and Translational Research in Population Health (ITR), 4200-450 Porto, Portugal; 5 Center for Study in Animal Science (CECA), ICETA, University of Oporto, 4501-401 Oporto, Portugal; 6 Associate Laboratory for Animal and Veterinary Sciences (AL4AnimalS), 1300-477 Lisbon, Portugal

**Keywords:** multiresidue method, LC-MS/MS, HRMS, immunoassay, food processing

## Abstract

Veterinary drugs are widely used to prevent and treat diseases. The European Union has forbidden the use of antibiotics as growth promoters since 2006. Its abusive use leads to the presence of antibiotic residues (AR) in foods of animal origin which is associated with antibiotic resistance. The monitoring of AR in food intended for human consumption is of utmost importance to assure Food Safety. A systematic bibliographic review was carried out on the analytical methodologies, published in 2013, for the determination of AR in foods of animal origin. The food processing effect in the AR detected in animal products is also addressed. However, there is a preference for multiresidues multiclass methods, i.e., methodologies that allow determining simultaneously different classes of antibiotics, which is still a challenge for researchers. The wide diversity of physico-chemical properties of these drugs is an obstacle to achieving excellent analytical performance for a vast number of molecules analyzed concurrently. New techniques in sample preparation continue to be developed in order to obtain a compromise between good recoveries and extracts without interferences (clean extracts). The most widely used analytical methodology for the determination of AR is liquid chromatography coupled with mass spectrometry. However, the current trend is focused on the use of powerful high-resolution MS detectors such as Time of Flight and Orbitrap with modern chromatographic systems. Cooking time and temperature control are the key processing conditions influencing the reduction of AR in foods.

## 1. Introduction

Veterinary medicines are substances or combinations of substances used to cure, prevent, or diagnose disease in animals. They are also known as medicinal products for veterinary use, veterinary drugs, or veterinary medicinal products (VMPs) [1].

The administration of veterinary drugs is mostly used for the prevention of diseases, such as in stressful conditions (e.g., transportation of animals) and overcrowded reproduction conditions [2]. Antimicrobials are substances that are synthesized or naturally created and are used to destroy or inhibit the growth of microorganisms such as bacteria, viruses, fungi, or parasites, especially protozoa.

Antibiotics are antimicrobial substances formed by or obtained from microorganisms that kill or inhibit the growth of other microorganisms [3]. Today, however, the word “antibiotic” is sometimes used to refer to the entire class of drugs as a synonym for the more general term “antibacterial” [4]. Antibiotics were first approved for use in livestock by the US Food and Drug Administration (FDA) in 1951 [3].

Global antibiotic consumption is projected to rise by 67% between 2010 and 2030, mainly due to the need for intensive large-scale livestock production that is substantially dependent on the application of antibiotics. This is also related to the increased demand for animal protein, especially in Asia [2]. As a result, the expected growth rate of antibiotics for countries like Brazil, South Africa, China, India and Russia is 99%, which is seven times the estimated population growth rate over the same period.

Many countries may not have access to antibiotic utilization statistics, and regulatory control may be limited. Many antibiotic groups/families that are used in humans are currently employed in the poultry, dairy products, cattle, and pork sectors.

A high percentage of antibiotic sales comes from large food-producing industries. For example, in the United States, 42% of sales are from livestock, with 39% from pig industries. Tetracyclines accounted for 66% of antibiotic sales for livestock in 2018, with a percentage of 13% for penicillins, macrolides at 8%, fluoroquinolones at <1%, lincosamides at 2%, aminoglycosides at 5%, cephalosporins at 1% and sulfonamides at 5% [2]. Antibiotic use in chicken, on the other hand, has reduced. In the United States, 92% of chickens sold did not receive medically important antibiotics on a regular basis in the year 2018.

Most changes in chicken occurred recently, with a drop of more than 70% between 2013 and 2017 [2]. The most often used antibiotics in pigs are tetracyclines and penicillins [5].

In recent years, under different environmental conditions, including soil plants, water and air, and the emergence and spread of drug-resistant bacteria, the pollution of veterinary antibiotics have been recorded [6].

Therefore, even at very low concentrations of veterinary antibiotics, there is a risk to human and ecological health, causing microbial resistance in the environment and leading to severe allergies or toxicity to plants and humans [7,8].

Children are the most vulnerable to veterinary drugs in the diet among global consumers. Furthermore, of the increased rate of consumption per unit weight, this group is regarded to be at high risk considering they are more vulnerable to veterinary drugs than grownups [9].

The introduction of antimicrobial agents to promote growth in animal feed has been banned by the EU since January 2006 through Regulation (EC) No 1831/2003 [3]. This, however, has not been enough because the presence of antimicrobial agents in animal products is still prevalent in countries such as North America, Australia, Brazil, and others ([4]). Naturally, this is a general problem, as bacterial resistance continues to grow with serious consequences for the global population.

Here, a comprehensive review of the antibiotics’ residues in animal-based food is presented, focusing on the most recent and valuable extraction procedures and analytical techniques for qualitative and quantitative analysis of multiresidues of different antibiotic classes. Special focus is devoted to methods that include the analysis of the aminoglycosides group. The legal instruments adopted by different countries/groups of countries to define the maximum residue limits (MRL) of these substances in foods are also compared.

The impact of food processing on antibiotic residues present in animal-based food is also thoroughly discussed, raising discussion about the pre-set residue levels in processed food and about the need for the development of methods for the detection of multiresidue antibiotics in processed food samples. Finally, the authors explore possible future advancements in the investigation of antibiotic residues in animal-based foods.

## 2. Legal Frame of Maximum Residue Limits (MRL) of Antibiotics in Foods of Animal Origin

In the EU, to maintain food safety, residues of veterinary medicinal products remaining post-treatment in foodstuffs of animal origin must be scientifically evaluated in compliance with Regulation (EC) n° 470/2009 of the European Parliament and of the Council of 6 May 2009 [10].

To limit human exposure, the organization’s Codex Alimentarius (Codex Guidelines for Establishment of a Regulatory Program for Control of Veterinary Drug Residues in Foods) [11], the European Union (EU) [10] and the FDA [12] established maximum residue limits for pharmacologically active substances, i.e., the maximum concentration of a pharmacologically active substance that may be allowed in food of animal origin [1].

**Figure 1:**
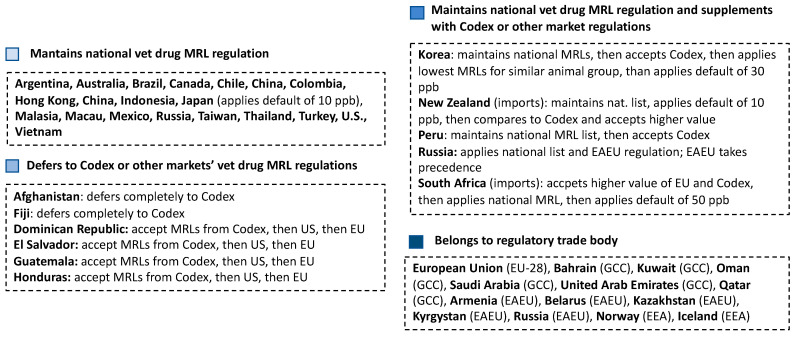
Veterinary drug MRL regulations used in countries around the globe. Adapted from the article “Veterinary Drug MRLs—A Global Perspective, by Ann Stevenson, Manager, Regulatory Data. Bryant Christie Inc”. European Union (EU), European Economic Area (EEA), Gulf Cooperation Council (GCC), Eurasian Economic Union (EAEU).

When importing or exporting animal products, the maximum residue level (MRL) of veterinary drugs should be considered. Regulations are often different among countries, leading to obstacles in economic transactions (Figure 1). For example, for a specific substance, chlortetracycline in swine muscle, the USA and Canada established an MRL of 200 µg/kg, but the EU has a more restrictive MRL of 100 µg/kg.

The European Union requires its members to implement an annual monitoring plan (official controls) to detect the illegal use (Group A, Council Regulation (EEC) 2377/9) or misuse of authorized veterinary medicines (Group B, Council Regulation (EEC) 2377/90) under the coordination of the Food and Veterinary Office (FVO) [13]. Furthermore, Commission Decision 2002/657/EC describes the guidelines for the validation of analytical methods and the expression of results [14]. The consequence is a necessity for a methodology to improve a higher sensibility, accuracy, rapidness and reliability. Another important subject is RASFF–Rapid Alert System for Food and Feed, which is a tool for ensuring the flow of information and allowing rapid response when threats to public health are found in the food chain. RASFF, founded in 1979, enables efficient information sharing among its members (European Commission, EU Member State national food safety authorities, ESA, EFSA, Liechtenstein, Norway, Iceland and Switzerland) and provides a 24 h service to ensure that urgent alerts are received and confirmed efficiently. Many food safety threats were avoided thanks to RASFF before they could have harmed European consumers [15].

## 3. Physico-Chemical Properties of Different Classes of Antibiotics

**Figure 2:**
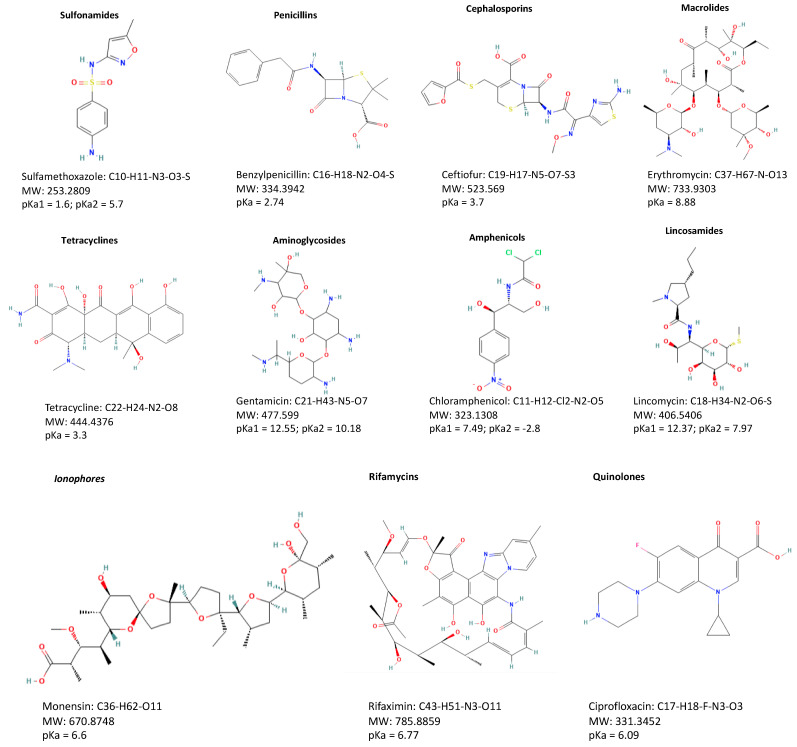

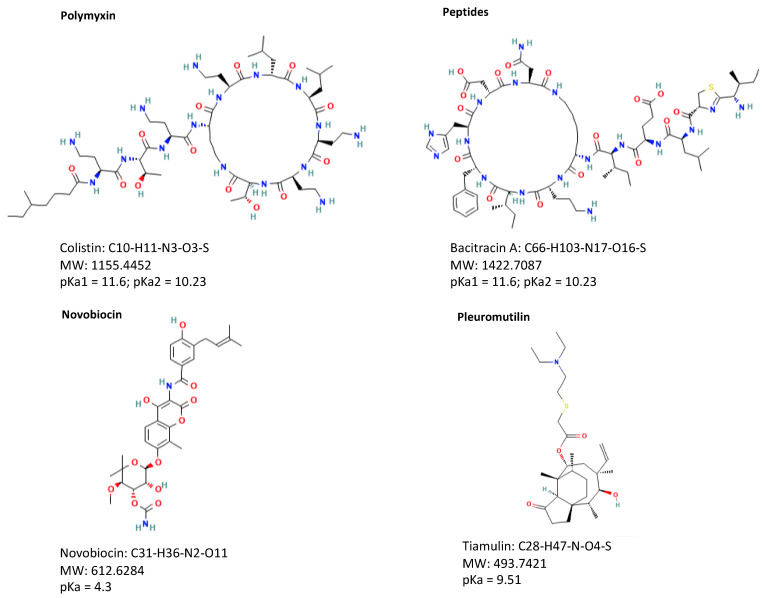
Structures of representative compounds from each class of antibiotics.

The different classes of veterinary drugs are directly related to different chemical structures that consequently give rise to different physico-chemical properties (Figure 2).

Table 1 compiles the classes of veterinary drugs most used in the EU according to the EFSA report on “monitoring of veterinary medicinal product residues and other substances in live animals and animal products” [16].

### 3.1. Sulfonamides

Sulfonamides (SA) are sulfanilamide derivatives that serve as the structural nucleus for many of the compounds in this class. They have varied pharmacological and bactericidal effects, as well as changing physico-chemical properties, depending on the attachment or replacement of different functional groups in the amido group or replacement in the amino group.

Despite their amphoteric nature, SA typically act like weak organic acids and are much more soluble in alkaline aqueous solutions than in acidic solutions.

The pKa value is 4.8–8.6 [17]. While a group of diaminopyrimidines (aditoprim, methoprim, pyrimethamine trimethoprim, ormetoprim) is commonly used alone, they are ineffective against bacteria, and resistance develops quickly. When separate SA are combined, however, a sequential blockade of microbial enzyme systems occurs, with bactericidal consequences. Trimethoprim/sulfamethoxazole (SMX) (co-trimoxazole), trimethoprim/sulfadiazine (SDZ) (co-trimazine), trimethoprim/sulfadoxine (co-trimoxine), and ormetoprim/sulfadimethoxine are examples of such potentiated SA preparations [18].

### 3.2. Tetracyclines

Tetracyclines (TC) occur naturally in three forms. Chlortetracycline (CTC), oxytetracycline (OTC), and desmethyltetracycline, some of which are semi-synthetic derived (tetracycline (TC), rolitetracycline, metalocycline, minocycline), doxycycline (DC), lymocycline, etc.). The elimination time can be further divided into short-acting (TC, OTC, CTC), medium-acting (desmethylchlorotetracycline and metcycline) and long-acting (DC and minocycline).

TCs are semi-synthetic products with a bulkier side chain than minocycline and are stable in dry powder form but not in aqueous solution, particularly at higher pH ranges (7–8.5) [17]. They are water-soluble, strongly polar compounds [19]. They are also poor bases, with pKa values ranging from 3.2 to 9.8 and a variety of chromophore groups. The class of tetracyclines has a good chelating capacity because positions C1 and C11 have two distinct ketone groups [20].

### 3.3. Penicillins

Penicillins, especially the β-lactam ring, are somewhat unstable and sensitive to light, heat, oxidizing and reducing agents, heavy metals and extreme pH. During sample preparation, they typically exhibit very low analyte stability [21].

Penicillins are acid and base sensitive, and their sensitivity varies depending on the nature of the side chain. Furthermore, the presence of nitrogen in the β-lactam induces a reaction with chemical substances, such as nucleophiles like methanol, which is increased by heating and acid catalysis [22]. The best application of β-lactam is in synergy with β-lactamase inhibitors. Cefoperazone with sulbactam or amoxicillin with clavulanic acid are good examples of combinations to increase the effectiveness of these classes of compounds. For instance, the most frequent β-lactamic antibiotics are ampicillin, cefapirin, cloxacillin, penicillin G and amoxicillin [23].

### 3.4. Cephalosporins

Cephalosporins have chemical and physical characteristics that are somewhat similar to penicillins however are more resistant to temperature and pH changes. Cephalosporins are a class of weak acids produced from 7-aminocephalosporanic acid.

The first-generation cephalosporins, including cephalothin (no longer sold in the United States), cephaloridine, cephradine, cefazolin and cephalexin, cephapirin and cefadroxil, are examples of molecules of this group. Cefoperazone, cefotaxime, ceftiofur, ceftriaxone, and many others, including cefovecin and cefpodoxime, are among the second-generation cephalosporins. Cefepime is a fourth-generation cephalosporin antibiotic [17].

### 3.5. Macrolides

Macrolides are a type of antibiotic that is commonly used in veterinary medicine to treat respiratory diseases or as a food additive to promote growth.

A macrolide is a complex mixture of antibiotics that varies in the chemical substitutions of the structure’s multiple carbon atoms, as well as the neutral amino sugars. For example, Erythromycin is predominantly erythromycin A, but types B, C, D, and E may also be present [24].

Macrolides are basic because they contain a dimethylamino group. While it is not soluble in water, it does dissolve in more polar organic solvents. In alkaline (pH 10) and acidic (pH for erythromycin) conditions, macrolides are often inactivated. Furthermore, because of their numerous functional groups, they can perform a wide range of chemical reactions [17].

### 3.6. Quinolones, including Fluoroquinolones

Quinolones share several similar functional groups that are important for their antimicrobial effect, despite the diversity of their ring structures. The carboxylic group at position three makes the compounds acidic. However, the 7-piperazinyl quinolones even have basic amino substituents. In solution, the 7-piperazinylquinolones are cationic, zwitterionic or anionic, whereas the opposite quinolones will solely be neutral or anionic. Thanks to the various form of substituents, quinolones have reciprocally rather totally different physical properties [25]. Some quinolones are eliminated unchanged (e.g., ofloxacin), some are partially metabolized (e.g., ciprofloxacin, enrofloxacin), and a couple are completely degraded [26]. Metabolites are typically active; enrofloxacin is de-ethylated to make ciprofloxacin.

Enrofloxacin is an antimicrobial drug that was developed specifically for application in veterinary medicine in the late 1980s. Enrofloxacin is a fluoroquinolone antibacterial agent of the third generation. This antibiotic is efficacious against a broad spectrum of infections in animals and is applied in the prevention or treatment of infectious diseases [27].

Administered orally in chickens, turkeys, pigs and cattle (through food, milk replacer and/or drinking water) or by injection to pigs or cattle parenterally [28]. Enrofloxacin is well absorbed, dispersed into tissues, and excreted in high amounts in the urine and feces after oral administration. One of the main metabolites of enrofloxacin is ciprofloxacin, which is metabolized in the liver [29].

### 3.7. Aminoglycosides

An aminocyclitol group characterizes aminoglycoside antibiotics, and a glycosidic bond connects the amino sugar to the aminocyclitol ring.

Gentamicin is a mixture of gentamicin C1 and C2, whereas neomycin is a combination of neomycin B and C as well as framycin [17].

Its solubility in water is improved by the presence of hydroxyl groups, whereas its solubility in fat is decreased by the presence of amine groups. These drugs’ pKas are usually between 8 and 10 [30].

### 3.8. Phenicols

Chloramphenicol is a straightforward neutral nitrobenzene derivative. It is extremely lipid soluble and can be employed as a free base or an ester [17].

The methylsulfonyl group of thiamphenicol and florfenicol replaces the nitrophenol group of chloramphenicol; florfenicol also includes fluorine molecules. These structural improvements can increase effectiveness and minimize toxicity, and fluorine molecules can reduce bacterial resistance in the case of florfenicol.

### 3.9. Lincosamides

Lincosamides (LCs) are a group of drugs very similar to macrolide drugs. The main chemical property that differentiates them from macrolides is an uncommon eight-carbon sugar. They are more soluble in salt forms of amino acid and sulphur-containing octose because they are monobasic (hydrochlorides and phosphates). They are capable of forming good salts with hydrochloric acid (HCl) [20]. The main members of the LS class are lincomycin and clindamycin [31].

### 3.10. Polymyxins

This group of polypeptide antibiotics includes polymyxin B and polymyxin E or colistin. Polymyxin has a synergistic effect when combined with enhanced SA, TC and other antibacterial agents. They also reduce the activity of endotoxins in body fluids and may be beneficial for endotoxemia [17].

### 3.11. Bacitracins

Bacitracin is a branched, cyclic decapeptide antibiotic. Bacitracin A is the most active and the main component of commercial bacitracin. Bacitracin has a broad range of actions, but it is most often used to treat Gram-positive bacteria. Since there is little resistance, bacitracin is typically used in conjunction with neomycin and polymyxin to broaden the antibacterial spectrum [32].

### 3.12. Novobiocin

Novobiocin is a narrow-spectrum antibiotic that, at higher concentrations, can have an antibacterial or bactericidal impact. It mostly inhibits Gram-positive bacteria but also a few Gram-negative bacteria. It works in tandem with tetracycline [17].

### 3.13. Tiamulin

One semisynthetic derivate of pleuromutilin is tiamulin. It consists of a tricyclic motilin core with a C-14 glycolic acid side chain, in which the C-21 keto group is essential for antibacterial activity. Studies have found that the side chain containing thioacetate in tiamulin has particularly strong antibacterial activity. It is possible to study the binding of pleuromutilins in more detail to examine compounds with a range of C-14 substituents. Although the tricyclic core has a hydrophobic interaction in the binding site, and the C-21 carbonyl group seems to be in the position of polar interaction, the rest of the C-14 side chain in the studied compound only forms a small contact. It does not appear to be involved in any major interaction [33].

### 3.14. Ionophores

Ionophores are fat-soluble molecules that carry ions through the membranes of lipid cells. They play an important role in improving the health and feed efficiency of livestock and poultry production. An ionophore that is usually utilized is Monensin. This compound is derived from Streptomyces and has the propriety of forming complexes with monovalent cations (including sodium and potassium). Monensin inhibits protein transport in cells, resulting in antibacterial and antimalarial impact. Monensin is commonly used in feed to avoid coccidiosis and increase feed production in the beef and dairy industries [17].

### 3.15. Rifamycins

Rifamycins belong to the family of antibiotics of ansamycin, whose name results from its basket-like structure containing aliphatic chains that connect the two ends of the naphthoquinone nucleus. The four structures of rifamycins presently authorized for use in the United States are rifampicin, rifabutin, rifapentin and rifaximin [34].

## 4. Extraction and Clean-Up Methods for the Determination of Antibiotic Residues

The complexity of animal matrices, presenting high fat and protein content, is a difficult challenge to reach good analytical performance. Extraction is essential for the correct detection and quantification of veterinary drugs. More than 70% of the entire research time is spent on sample preparation [22].

In general, extractive procedure techniques for the analysis of antibiotics in food have a few stages that can be repetitive, expensive, and limit the degree of adequate recuperation for certain drug groups [35]. Most sample preparation procedures have been built over the years. In the last decades, there has been a tendency to move from individual analyses of different classes of antibiotics to multiclass/multianalyte methods. Developments in sample preparation include recommendations for using smaller sample sizes, minimizing the use of organic solvents (sensitivity to ecological concerns), common extraction methods for multiclass compounds, and the potential for automation and/or high throughput [4].

Each class of antimicrobials has a unique structure, and this influences their activity and proprieties, including solubility, stability and polarity [21]. As a result, developing a single approach for studying various antibiotic groups is difficult and requires a compromise on the optimization of the procedure for individual classes. For example, SA has good solubility in polar solvents like acetonitrile (ACN), MeOH (methanol), acetone and chloroform. Jie et al. [36] determined 17 SA in porcine tissues (muscle, liver and kidney) using three different sample preparation methods. Two of them used new materials, including Oasis PRiME hydrophilic-lipophilic balance (HLB) and Enhanced Matrix Removal for Lipid (EMR-L), and the third one was the conventional solid–liquid extraction (SLE) with n-hexane. The results showed that EMR-L and HLB are recommended for pre-treatment of samples with high lipid content. In SA research, SLE demonstrated lower recoveries and higher matrix effects as compared to other sample preparation methods [36]. A method for multiresidue and multiclass quantification of antimicrobials in pangasius fillets was reported by Bortolotte et al. [37]. A quick, effective, and fast extraction procedure for SA using ACN and 0.1M ethylenediaminetetraacetic acid (EDTA) solution yielded good results: recoveries of 86.2% to 115.7% and CCα of 104.7 to 106.2 µg/kg [37]. The use of ACN with 0.1 M EDTA solution has been reported for extraction of antibiotics, including 14 SA, in piglet liver [38]. Recoveries ranged between 93% to 105%, and CCα values were between 107–134 µg/kg. During sample preparation of food products such as honey, an acid hydrolysis step is generally performed to release SA from the sugar-SA complex, increasing the recovery of the analytes (ob. cit [20,39,40,41]).

TCs are strongly polar, water-soluble compounds that chelate with metal ions [19]. Since under severe pH settings with strong acids and alkalis, TCs can degrade by isomerization, dehydration, epimerization, and other mechanisms. The proclivity of metal ions to form chelation complexes and matrix protein binding can cause problems in research [22]. Many authors have stated that using a complexing agent such as EDTA salts increases extraction performance with or without the addition of McIllavin buffer [42,43,44,45,46,47,48,49,50,51,52].

The primary techniques utilized for the extraction and clean-up of TCs from such matrices are solvent extraction (liquid-liquid extraction, LLE or SLE), solid phase extraction (SPE), ultrasonic-assisted extraction (UAE), pressurized liquid extraction (PLE), matrix solid-phase dispersion (dSPE), and molecularly imprinted polymer solid-phase extraction (MIP-SPE). Reddy et al. [45] studied the quantification of five TCs and their epimers in matrices such as seafood-based products, fish, meat, infant formulae, dairy ingredients and fats by LC-MS/MS. This method’s theory was based on LLE with ACN and an aqueous EDTA solution, proceeded by a freezing stage to facilitate phase separation at low temperatures. Following hexane degreasing, the sample extract was evaporated and reconstituted before being injected into the LC-MS/MS system. The target screening concentration (STC) for the five TCs and epimers is 50 µg/kg. Two independent laboratories, in the validation, included a total of 93 samples, and the result of the rates of false negatives and false positives for all compounds were 0%.

Optimization of a modified QuEChERS (quick, easy, cheap, effective, rugged, and safe) method for the determination of TC in fish muscle by Ultra High Performance Liquid Chromatography tandem Mass Spectrometry (UHPLC–MS/MS) was developed by Grande-Martínez et al. [42]. The experimental method was utilized to optimize the extraction step’s parameters, such as the amount of sample, EDTA-McIlvaine buffer, and the volume of extraction solvent. The key process parameters (such as the lowest matrix effect and the dSPE purification phase parameters) were optimized to achieve better results in terms of recovery and accuracy. The optimal extraction conditions for 1 g fish meat were 2.2 mL EDTA-McIlvaine buffer, 5.0 mL ACN and 1.25 g (NH_4_)_2_SO_4_. The most critical and important parameter was the volume of the EDTA-McIlvaine buffer. Citrate and phosphate of pH 4.0 promote the displacement of the balance to the zwitterionic form of TC, thereby increasing their solubility. Adding chelating agent EDTA can get proper TC extraction. The recovery rate of five TCs (OTC, metcycline, tetracycline, CTC, DC) is between 80% and 105%. Chiesa et al. [44] used HPLC-MS/MS to compare antibiotics in urine and muscle samples from food chain animals. The analytes were extracted using 5 mL of McIlvaine buffer (pH 4.0). Trichloroacetic acid (TCA) (100 mL, 20% w/v) was used to precipitate the proteins, and 2 × 3 mL of n-hexane was used to decant the supernatant. The n-hexane layer was discarded. Using Oasis HLB cartridges, the sample was further purified and extracted. In muscle tissue, the CCα ranged from 0.95 to 10.1 ng/mL, and the CCβ varied from 1.13 to 11.0 ng/mL.

Penicillins (β-Lactams) (PNs) have been demonstrated to be volatile, polar, and particularly heat sensitive (ob. cit. [20,53]).

In addition to their physico-chemical properties, β-lactams are very complex and difficult to extract in food analysis, either in individual or multiresidue methods. Van Holthoon et al. [54] devised a derivatization reaction to avoid the deterioration of penicillins during extraction. Their procedure consisted of precipitating milk proteins with acids and cleaning the supernatant in an SPE cartridge [55]. Recently, newer methods for β-lactams in food have been used, such as PLE, dSPE, liquid membrane extraction and magnetic MIP (ob.ci., [31,56]). Jank et al. [57] extracted 14 β-lactams in milk by a simple and rapid process of LLE with ACN. They obtained good results with recoveries between 67–108%, except for amoxicillin (AMX) (58%). Sun et al. [58] used ACN in LLE to establish a comprehensive study of AMX, its main metabolites, and ampicillin in eggs. Due to their amphoteric nature and strongly polar properties, AMX, AMX metabolites, and ampicillin are difficult to chemically analyze. In this technique, the authors adjust the pH of the extract to 6.74 with ammonium acetate to avoid the degradation of the target compounds. They reported recoveries of 80–98%, LOD between 0.1–0.6 µg/kg, LOQ from 0.3 to 0.8 µg/kg, CCα between 11.1–11.15 µg/kg, and CCβ with values between 12.1–13.0 µg/kg [58].

Macrolides (MAs) are non-polar molecules. The better extractive process for this type of class is in basic conditions. Due to their instability under acidic conditions, acidic solvents are not used in the extraction stage or mobile phase preparation [20]. Thompson and van den Heever [59] proved that in an acidic aqueous solution, erythromycin mainly produces anhydroerythromycin after degradation. It was confirmed that three degradation products (anhydroerythromycin, erythromycin, enol ether and an unknown but suspicious related isomer) with a molecular weight of 715 Da are formed in honey. However, the use of buffers for MA extraction from chicken tissues has been mentioned. McIlvaine–EDTA buffer was able to extract the MAs (tylosin, spiramycin, tilmicosin) in fortified samples with recoveries from 85–120% at 200 μg/kg ranges [60]. Lan et al. [61]. reported a method where MAs were extracted from chicken samples with 10 mL of ACN/MeOH (95:5, v/v) and 200 μL of 0.1 mol/L EDTA solution with good recoveries 82–101%. Another study showed that Tris buffer is used to determine lincomysin and five MA residues in honey at the same time (ob. cit., [20,62]). A procedure for analyzing MA and LS antibiotic residues in muscle from cattle, poultry swine and bovine milk, was developed and validated. ACN was reported efficient for the simultaneous determination of MA and LS antibiotic residues. The milk extraction method entailed the addition of 4.0 mL of ACN, divided into three aliquots of 2.0 mL, 1.0 mL and 1.0 mL to promote precipitation of milk proteins and resulted in recoveries ranging between 60% to 73%. Muscle samples extracted with ACN (2g: 10.0 mL) were homogenized in a mechanical mixer, resulting in recoveries from 69% to 107% [31].

ACN was also successfully used to extract LS in multiresidue studies, with good recoveries. Hu et al. [63] published a review article focusing on different techniques for extracting and quantifying MA based on a bibliographic survey from 2010 to 2020. They concluded that SPE had become the most popular technique because of its superb extraction performance and high recovery. It is vital to choose an appropriate SPE column based on the matrix and the analytes of interest. Compared with commercially available adsorbents (Oasis HLB, C18, MCX columns, etc.), new materials (such as MIP and magnetic materials) have huge potential applications in SPE adsorbents due to their high selectivity, stability and durability [63].

The class of quinolones (QN) is highly soluble in polar organic or hydro-organic solvents. Organic solvent extraction (ACN or MeOH) is successful for many veterinary drug residues in foods, but highly water-soluble drugs, such as QN, are recovered with low yields due to low extraction efficiency and the existence of significant matrix effects [64]. Therefore, many methods for determining quinolone residues in a single matrix, such as chicken tissues, poultry muscle, and eggs, have been established [65,66,67]. They often employ an LLE and an SPE to pre-concentrate and clean-up the extracts. Some papers reporting SPE methods indicate a preference for Oasis Prime HLB. This type of column provides more effective removal of matrix interference, especially phospholipids, and the fact that it does not require preconditioning or equilibration results in faster methodologies compared to conventional SPE [20,68,69]. For example, Annunziata et al. [69] established an extraction method for 11 QN in eggs and muscle using a mixture of MeOH and metaphosphoric acid 1% solution (40:60, v/v) with the internal standard norfloxacin d-5. The clean-up followed the lines of other authors, with HLB [64,68]. They reported mean recoveries for all QN ranging between 91 and 107% for muscle and 95 and 105% for eggs. In general, the results in this class of antibiotics depend on the solvent(s) applicated for the extraction procedure and precipitation of proteins; for example, 0.1% formic acid (FA) in acetonitrile/water (90:10, v:v) was used by He et al. [68] and a solution of EDTA Mcllvaine buffer, 0.1 mol/L, pH 4.0 was used by Lu et al. [64] for QN and sulphonamides analysis (Table 2).

Aminoglycosides (AG) belong to a very selective class within antimicrobial substances. The reason is associated with their polarity and functional groups, mainly amino and hydroxyl. In addition, they have a high affinity for protein binding and interact with the complex metal ions found in biological matrices [22]. As a result, TCA, whether pure or combined with other solvents, is the best solvent for AG extraction since it aids in matrix deproteinization [82]. This acidity and its strong ionic strength allow the extraction. However, a clean-up with SPE is required for the purification of the extract since the loaded solution presents interference [22,83,84]. For all these reasons, the inclusion of this class of antibiotics in multiclass/multianalyte foodstuffs is a challenge. In our bibliographic survey, few multiresidue studies, including AG analysis, were found. One of the most recent research studies in this field evaluated 76 veterinary pharmaceutics from 13 classes of antibiotics, including AG [72]. Because of the complex matrices and low levels of analyte concentration, these authors discovered that the extraction technique used for food and environmental samples is frequently substantially more extensive. The process incorporates a two-step extraction technique, ACN extraction accompanied by the acidic aqueous buffer, and then the determination of hydrophilic interaction tandem liquid chromatography-mass spectrometry (HILIC-MS/MS), which was performed in a single chromatographic method of confirmation and quantification.

Further cleanup with SPE was performed using polymeric SPE cartridges [72]. However, due to their high selectivity for aminoglycoside antibiotics, MIP-based materials appear to be the most promising. Furthermore, with the variety of separation modes accessible and compatibility with many different types of selective and sensitive detectors, liquid chromatography appears to be the most successful analytical platform [82]. An important advancement could be the employment of a variety of biosensors, such as electrochemical aptasensors, in a way similar to the holistic methods used in electronic noses and tongues, utilizing their partial specificity with multivariate statistical analysis and machine learning algorithms [82].

LLE, either alone or in combination with SPE, is a common method for analyzing amphenicols. Many individual solvents, like ethyl acetate, or mixtures, also associated with FA or phosphate solution, have been reported for amphenicols’ determination. Defatting requires hexane or isooctane, which can be mixed with ethyl acetate. To further remove excess water from the extract, chloroform may be applied to the mixture. ACN and hexane, and ACN and chloroform, are other mixtures also used in the analysis of amphenicols [76,85].

The Lincosamides (LS) class of veterinary drugs is weak and basic in nature. Few single analytical procedures are reported for these antibiotics because, normally, they are included in multiclass methods [31,43,86]. Antibiotics like polymyxins (polypeptides) have a molecular weight ranging from 1000 to 2000. The extraction methods use low pH extraction to release the protein analytes in the matrix. In order to obtain good recoveries, studies suggest that a low pH during the extraction procedure is required. On the other hand, this may lead to partial degradation of some analytes; therefore, it is important for stability at low pH since the extract remains in a low pH environment for a prolonged period (between extraction and SPE). It has been reported that the low pH of the extraction solvent (HCL) is likely to cause degradation of colistin but that acidified methanolic extraction solvents provide good recoveries for bacitracin, colistin A, colistin B, polymyxin B2 and polymyxin B1 polypeptides [87]. To minimize the amount of interfering substances, the extraction process usually involves an aqueous acid solution, MeOH or ACN, in different amounts accompanied by a reversed-phase SPE (primarily with polymeric sorbents) [88]. Polypeptides have similar physical and chemical properties as the major components present in meat. It is, therefore, not easy to separate them from the bulk of the matrix [87]. In addition, an extra critical factor is its high atomic weight, which requires the utilization of mobile phases that are specifically designed for peptides, like trifluoroacetic acid (TFA) or TCA, which is extremely disadvantageous for other molecules that may be investigated simultaneously, taking into account that these modifiers may cause potential ion suppression issues [22].

Other antibiotics, such as tiamulin, rifamicine and ionophores, are typically included in multiresidue methodologies. Schlüsener et al. [89] studied a small group of different antibiotic classes–macrolides, ionophoros and tiamulin, by LC-MS/MS. The procedure includes an extraction with ethyl acetate followed by purification by SPE with glycol filling [89]. Recently, De Baere et al. [90] published an article on 8–hydroxy-mutilin, which is a marker residue of Timilin for rabbit tissues (muscle and liver). The extraction solvent used is a mixture of acetone/HCl (50/50, v/v), tiamulin, and its metabolites are hydrolyzed to 8-hydroxy-mutiline in a basic medium at 45 °C. For LLE, acetic acid is used in an acidic medium of ethyl acetate. The recovery rates of muscle and liver were 66.2% and 75.5%, respectively. Dubreil et al. [52] used LC-MS/MS to establish and validate a screening system for 75 antibiotic residues in aquaculture products and meat. Before being expanded to aquaculture products, the method was validated for three distinct species: chicken, cattle, and pork. The strategy used was an LLE with 800 µL of water and 8 mL of ACN for each 2 g sample [52]. The validation level ranged between 50 to 400 µg/kg.

Multiple extraction procedures for determining antibiotic residues in foodstuffs have previously been published and discussed. The detailed explanation of the basis of each technique is not under the scope of this review and has already been discussed in detail by several authors [9,20,22,91,92,93,94,95]. In this line, we present in Table 3 a brief summary of the main extraction techniques used in the study of veterinary drugs in foods within the umbrella of Green Chemistry.

## 5. Multiclass Multiresidue Methods

Many single-class methods are reported in the literature, but multiclass multiresidue approaches are hampered due to the sample preparation stage that remains challenging for most researchers (Table 2). For instance, the existence of high protein and fat content in some types of food results in a more complex purification of the extract; large differences in the polarity of the analytes under study make it difficult to choose solvents in the extraction stage, and the presence of tissue enzymes that can cause the degradation of some analytes [51].

In the sample preparation stage, multiclass multiresidue methods involve two fundamental steps, the removal of interferences in the sample and the retention of the target compounds. Interference removal generally requires the use of more common and less restricted sample extraction methods, such as SLE or QuEChERS extraction.

SLE is commonly used to extract analytes from a sample matrix using ACN [52], a mixture of water/ACN, MeOH/ACN or buffer. These extraction procedures are based on the extraction of many target analytes and the prevention of labile analyte (s) from deteriorating. In addition, the rapid determination of an extensive number of analytes is characteristic of these analytical applications. However, the fact prevails that the use of the water/ACN mixture does not eliminate certain proteins that stay soluble, resulting in a more complex extract [51]. Since aqueous solvents are commonly used for polar compound extraction, the ACN/water ratio is usually optimized. Chen et al. [96] investigated the ideal extraction solvent. They concluded that the water/ACN mixture (90:10, v/v) was very efficient for the study in milk, tissues and eggs of 12 families of veterinary antimicrobial agents (β-lactams, quinolones, sulfonamides, macrolides, lincomycins, tetracyclines, nitroimidazoles, quinoxalins, polypeptides, chloramphenicol antibacterial synergists and others). Mixtures of water/buffer are reported by many authors. Zheng et al. [50] and Wang et al. [97] used different mixtures to extract families like tetracyclines, sulfonamides, polypeptides, quinolones, macrolides, lincomycines and others. However, special attention shall be given to fat-soluble compounds, macrolides or ionophores that cannot be largely recovered using this approach.

The methodology QuEChERS use the extraction solvent, ACN. For the induction of the separation of phases, the addition of inorganic salt is used [49,51,98]. These difficulties were solved with the use of ammonium sulfate salt for phase separation and cleaning, including the aqueous and ACN phases. Another issue is that, despite the fact that the QuEChERS approach is very versatile and several improvements have been suggested, the research still emphasizes that conditions must be optimized to satisfactorily restore each antibiotic group (type and quantity of salt, SPE volume, etc.) [97]. Khaled et al. [99] investigated the solid phase microextraction (SPME) process compared to two sample preparation procedures (solvent extraction and QuEChERS). Compared with the 30% analyte in QuEChERS and the 42% solvent extraction in beef tissue, SPME showed a much smaller matrix effect, and only two compounds showed a significant matrix effect. All methods can be used in the chicken matrix to obtain excellent accuracy and precision results, with more than 91% of the analytes falling within the 70–120% concentration range.

Cleanup techniques in multiclass multiresidue include SPE [50,51,80,81], hexane defatting [47,50,74,75], lipid freeze-out [47,96], C18 [80], Zr (Zirconium) sorbent or combinations [100]. However, these matrix cleaning methods result from poor selectivity, disappearance of analytes, and inability to properly remove lipids, as well as methods that can be extensive. The SPE technique is used to isolate target compounds from complicated matrices and to remove unnecessary matrices prior to eluting the target analytes. This represents a challenge in the development of multiclass and multiresidue methods because the mixture of analytes and the adsorbent has different binding affinities [51]. Since SPE sorbents have limited contact mechanisms, using SPE for multiresidue screening of components with different physico-chemical properties has some limitations [101]. Pugajeva et al. [101] evaluated two methods of extraction for more than 140 pharmacologically active substances in meat. The first procedure records a solvent extraction technique, the second an SPE technique (Phree^TM^ phospholipid removal column or Strata X SPE column preconditioned with ACN) and the third a dSPE technique. Due to analyte losses, the authors concluded that only the dSPE procedure, which used primary secondary amine (PSA) for cleaning, was unsuitable for the study of a wide variety of compounds. The best-performing results were obtained using two different extractions: solvent extraction with a freezing step and SPE using the Phree^TM^ phospholipid removal column or a Strata X column [101]. PRiME HLB is one of a new generation of sample preparation sorbents that have recently been created (process, strength, improvement, matrix effect, user-friendliness) [79]. Compared with traditional SPE adsorbents, Oasis PRiME HLB has many advantages. These benefits include the opportunity to remove pre-treatment and sorbent balance, resulting in a smoother workflow than conventional SPE materials. Another advantage is to increase the column life and reduce contamination of the source of MS by reducing interference from the matrix, for example, phospholipids [68]. Several authors used this dSPE column in different types of samples [68]. Zheng et al. [50] reported recoveries in the range 69.8–103.3% in duck and meat for 16 antibiotics belonging to seven different groups (penicillins, SA, macrolides, lincosamides, TC’s, trimethoprims and fluoroquinolones,). To avoid complexation of the TC and macrolides with metal ions, which could lead to low recoveries, the SPE procedure used 0.1 M Na2EDTA in combination with 2% TCA and n-hexane in combination with Oasis PRiME HLB cartridges. Turnipseed et al. [102] used the Oasis PRIME HLB cartridge to remove phospholipid interferences to identify veterinary drug residues such as fluoroquinolones, dyes, avermectins, quinolones, and aminopenicillins in fish. They obtained a good performance for the range of 1–200 μg/kg, depending on the analyte. Wang et al. [97] developed a multi-class approach (11 drug groups, 125 compounds) for analyzing veterinary drug residues in milk. To precipitate milk proteins, veterinary drugs were extracted using a modified salting out supported liquid extraction (SOSLE) method that requires the use of extraction buffers (oxalic acid and EDTA, pH 3) and ACN (salting out ACN/water phase. The authors used ammonium sulfate for separation and a polymer reversed-phase adsorbent (OASIS column) for SPE. Regarding method sensitivity, 71.6% could be detected and quantified below 1.0 g/kg concentration level, and 83.3% below 5.0 g/kg concentration level. The method was less sensitive to and β-lactams, penicillins, TC, with the lowest concentration levels ranging from 20.0 to 60.0 g/kg. In 2015, EMR-Lipid, a new sorbent for matrix removal, was released for dSPE clean-up of sample extracts with high lipid content. dSPE is a technique that mixes the sample on a solid support that can be based on silica and/or polymer. The rupture of the sample matrix is then achieved. Then, a cartridge is topped up with this combined mixture, and the analytes are eluted with appropriate solvents (Manimekalai et al. [20]). The selective interaction that occurs with the EMR-Lipid sorbent and the linear hydrocarbon chains of lipids make this type of clean-up ideal for future matrix interference elimination procedures. They were launched in 2017, and unlike the dSPE technique, they are manufactured in a cartridge, which facilitates and contributes to the reduction of the analysis time. As the name implies, they are favorably used in matrices with high fat content in the analysis of pesticides and multiclass and multiresidue veterinary drugs with excellent results [51]. Zhao et al. [51] validated an LC-MS detection system for the determination of multiclass multiresidue veterinary drugs using lipid removal clean-up cartridges, EMR-Lipid, for various meat matrices (muscle, kidney, and liver). They concluded that EMR-Lipid cartridge clean-up offers high matrix co-extractive removal and decreases matrix ion suppression on target analytes. The quantitative analysis results revealed that in all five meat matrices, more than 90% of studied veterinary drugs (39 compounds) generated adequate recoveries, and more than 95% of compounds provided excellent reproducibility. Another example is the application of EMR-Lipid in the comparison of two sample processing methods for the multiresidue analysis of veterinary drugs in milk using UHPLC-MS/MS [79]. The first procedure was an LLE with ACN (2% FA) and a clean-up in the PRiME HLB cartridge. The second procedure consisted of an ACN (5% FA) extraction and cleaning with EMR-Lipid dSPE (EMR-Lipid dSPE and EMR-Lipid Polish tubes). The authors concluded that HLB PRiME yielded the best performance, achieving negligible matrix effect for 65% of the compounds (66 compounds), while EMR-lipid yielded 43% mild matrix effect and 23% medium matrix effect.

MIPs as selective sorbents in SPE, known as MIP-SPE, is a new clean-up technique for complex matrices. MIP-SPE can compete with conventional SPE phases and immunosorbents in terms of selectivity, stability, and price. The best results for selective extraction of difficult samples, such as food, are obtained by combining specific cavities within the MIP with the proper selection of packing, cleaning, and elution solvents that promote specific interactions within the cavities [4]. Bixia et al. [73] used LC-MS and MIP-SPE to determine 11 aminoglycoside residues in milk, honey and pork. The aminoglycoside antibiotics were extracted using a solution containing 0.4 mmol/L EDTA-Na2, 10 mmol/L potassium dihydrogen phosphate and 2% TCA. The reagent for honey samples was 50 mmol/L potassium dihydrogen phosphate. MIP-SPE cartridges were used to purify the extracts. LODs ranging from 2–30 µg/kg and LOQs ranging from 7–100 µg/kg were obtained. The average recovery rate ranged between 78.2 and 94.8%. In another paper, Savoy et al. [103] identified a method for screening 14 aminoglycosides in foodstuffs of animal origin. The extraction of aminoglycosides is made in an acidic aqueous solution, EDTA 0.5% in water, TCA 2% in water. After, they were centrifugated, then diluted with an alkaline buffer of 80 mM ammonium carbonate, and finally purified by MIP-SPE. MeOH and 50 mM potassium phosphate solution (pH = 7) were used to condition the SupelMIP^®^ SPE-aminoglycoside cartridge. After loading a volume into the cartridge, it was washed with water, water: ACN (60/40, v/v), and dichloromethane/MeOH (50/50, v/v). Finally, the analytes were eluted with 30 mM heptafluorobutyric acid (HFBA) in ACN/water (25/75, v/v). For all substances, the screening method yielded a below 3% false-negative and false-positive rate.

Regarding aminoglycosides residues in foodstuffs of animal origin, the specificity of their physico-chemical proprieties, such as their high solubility in water, moderately soluble in methanol and insoluble in non-polar organic solvents [82], makes the simultaneous determination of aminoglycosides and other drugs in a single method process extremely difficult. The literature has very few articles for the detection of multiclass multiresidues of veterinary drugs in which aminoglycosides are included. In fact, two recent works have carried out the analysis of aminoglycosides. Dasenaki et al. [72] have developed a method for the analysis of 76 veterinary pharmaceuticals from 13 classes including aminoglycosides in bovine muscle, by hydrophilic interaction LC-MS. The method combines ACN extraction, an acidic aqueous buffer extraction, 10 mM ammonium acetate, 0.4 mM EDTA, 1% NaCl (w/v) and 2% TCA (w/v) in H_2_O with HILIC–LC-MS/MS determination in a unique chromatographic run. The study used an analysis of ten different antibiotic families (including aminoglycosides). As far as we know, this is the first work to use apramycin and neomycin in a multiresidue method. The extract was cleaned in an OASIS HLB (200 mg, 6 mL) column pre-conditioned with MeOH and water. The analytes were eluted with 1 mL 10% (v/v) FA aqueous solution and 3 mL ACN. The eluate was collected and combined with 1 mL of ACN extract. They obtained LOQ in the range of 0.03–17 µg/kg, CCα of 2.2–1151 µg/kg, and CCβ between 2.4–1302 µg/kg. Since aminoglycosides are not efficiently ionized in low water content solvents, the authors documented generating a final extract adequate for aminoglycosides determination and HILIC-compatible (>60% ACN). Reagents such as sodium chloride, EDTA, and TCA were required for the successful extraction of aminoglycosides, as well as other polar drugs such as sulfaguanidine, penicillins, and thyreostats. The pH value of 6.5 was considered a good value for both aminoglycosides and other classes of veterinary drugs. Lehotay and Lightfield [77] developed a method in bovine tissues with a large scope of drug residues (175 compounds), including the aminoglycosides class. In opposition to the traditional approach, in this methodology, the ion pairing (IP) reagent was added to the final extract and not in the mobile phase. They discovered that adding the IP reagent to the final extracts reduces the number of unwanted salts added to the MS source, reducing the need for subsequent maintenance interventions in UHPLC-MS/MS. Two different preparation sample methods were reported, one for the multiclass multiresidue methods and the other for the aminoglycosides (Table 2). Aliquots of each of the sample preparation methods were combined into a 1 mL autosample vial. Then, the heptanesulfonate IP reagent solution was added. The study was carried out on three matrices: kidney, liver and muscle. Recoveries ranged from 70–120% in 79–84% of the analytes. In muscle, the recoveries of aminoglycosides were significantly lower. The poor results are explained due to the higher fat content in the muscle extracts that clogged the DPX (weak cation exchange cartridges) tips during sample preparation. Furthermore, Lehotay and Lightfield [77] concluded that for drug analytes, fine, moderate, or bad recoveries and RSDs were observed, as previously mentioned.

In recent work, the authors Desmarchelier et al. [104] describe a new validation for a screening methodology of 154 veterinary drugs by LC-MS/MS. The aim of this procedure is to analyze multiclass multiresidues in different matrices of animal origin; fresh meat, seafood, as well as fish powdered samples (milk, meat, fish, and eggs). The analysis of the different classes of veterinary residues is divided (split) into four procedures (“streams”). The first stream (A) comprises 58 antibiotic and anti-inflammatory, as well as six antiparasitic residues. The beta-lactam class is included in the second stream (B) with 23 analytes, and finally, the last two streams (C and D) evaluate 14 aminoglycosides and 10 tetracyclines with their epimers.

These analytical methodologies apply different extractions and clean-up techniques depending on the characteristics of each class of analytes. For streams A and B, determination by the Acidic QuEChERS–like and the Alkaline QuEChERS–like are applied to the study of 129 analytes. The extraction of analytes of stream C is performed through the Molecularly imprinted polymer, while stream D uses liquid-liquid extraction for the study of the 10 tetracyclines and epimers.

This methodology is an AOAC Official Method (2020.04, “Screening of 154 Veterinary Drug Residues in Foods and Animal Origin Using LC-MS/MS, First Action 2020) very promising with probabilities of detection at Screening Target Concentration (STC) > 94% and at the blank percentage of <4%.

### 5.1. Detection Methods for Multiresidues of Antibiotics

- *Chromatographic methods: from HPLC to UHPLC*

Numerous advantages are related to the use of UHPLC in multiresidues analysis, such as reduction in analysis time, better resolution and detectability, the economy of stationary and mobile phase, small volume sample, ease of transferring a method developed by HPLC to UHPLC, a wide variety of columns and equipment available and less waste generation compatible with the principles of Green Chemistry. As a result, combining the two methods to form UHPLC-MS/MS can provide considerable benefits: a greater scope of target compounds, increased recoveries and greater sensitivity in UHPLC-MS/MS compared to individual methods. The use of UHPLC to separate matrix co-extracts and target compounds, as well as the selectivity provided by MS/MS detection, reduces interferences from matrices such as lipids and proteins [105].

- *Chromatographic methods: analytical columns*

In multiresidues analysis, the most common analytical columns are C18 reversed- phase columns. For instance, Kong et al. [106] used a C18 column (2.6 μm particle size) for the separation of more than 120 compounds by Orbitrap MS in the screening method. They validated the method with a screening level in the range levels of 1–50 µg/kg. Anumol et al. [35] compared the use of UHPLC-QqQ and QToF to quantify veterinary drug residues in animal tissues. The analytical column HSS T3 (1.8 μm particle size), which is ideal for use with 100% aqueous mobile phase, was used for the UHPLC-QqQ system, while the C18 column (1.8 μm particle size), which is particularly effective for the separation of acidic, basic, and other highly polar compounds by reverse-phase LC, has been applied to UHPLC-QToF system. Both instruments produced very similar results. The interference resulting from the nature of the matrix in QQQ limits the levels of quantification for targeted analytes, but the QToF allowed to detect and quantify the veterinary drugs under study within the regulatory limits of interest in this analysis. The partition of polar analytes between a layer of water-enhanced dissolvable close to the outside of the sorbent and the somewhat more hydrophobic eluent is fundamental to the HILIC retention technique (typically ACN). Other separation processes, such as hydrogen bonds, ion exchange and dipole–dipole interactions, are used in addition to the hydrophilic partition. HILIC columns are compliant with MS detectors and, compared to conventional reversed-phases, have higher sensitivity without the need for the addition of fluorinated ion-pairing reagents solving the troubles of ion suppression and prolonging the period of LC system maintenance [88]. The sulphoalkylbetaine groups in these zwitterionic columns have distal negative charges that control interactions with positively charged aminoglycosides. For example, they are used by Savoy et al. [103] and Dasenaki et al. [72]. Lehotay and Lightfield [77] used an HSS T3 (1.8 μm particle size) analytical column to simultaneously analyze aminoglycosides and many other classes of drug residues. In the final extracts, they employed reagent ion pairing (IP) to limit the number of unwanted salts added to the MS source (Table 2 and Table 4).

- *Chromatographic methods: the selection of the mobile phase*

Concerning the mobile phase (Table 2 and Table 4), it seems evident that the most common mobile phase used is a gradient with an aqueous solution with 0.1% FA and ACN with 0.1% FA [51,57,58,76,106,110]. Several authors reported that the sensitivity of drugs was higher when ACN was used as the organic mobile phase rather than MeOH, as reported by Aguilera-Luiz et al. [111,112]. However, the addition of FA led to an improved ionization efficiency as compared to that of acetic acid in water [96]. Nevertheless, other authors [47,72,98] used different combinations of aqueous solution with MeOH as an organic solution to improve the analytical performance of the most polar compounds, like aminoglycosides, cephalosporins and β-lactamics.

- *Chromatographic methods: from tandem mass spectrometry to high resolution mass spectrometry*

LC coupled to tandem mass spectrometry (LC-MS/MS) has become the most commonly used detection technique for veterinary drug analysis in foodstuffs because of its excellent sensitivity and selectivity [43,44,47,48,52,68,72,75,76,96,105]. The new trend is aimed at efficient high-resolution mass spectrometric detectors (HRMS), such as Time-of-Flight (ToF) [36,38,49,74,80] and Orbitrap [86,88,97,101,106].

HRMS has advanced due to the availability of more reliable, sensitive, and selective instruments. HRMS offers significant advantages over traditional unit mass resolution tandem mass spectrometry. A sequence of full-scan spectra, for example, provides better information about the sample as well as the capacity to determine compounds without prior compound-specific tuning [88]. Moreover, HRMS can measure the m/z ratio of each monitored ion up to many decimal numbers so as to obtain accurate masses rather than nominal masses [102]. In comparison to conventional tandem mass spectrometry with the unit mass resolution, HRMS allows for various acquisition studies as well as a retrospective analysis of initially analyzed samples. HRMS has the fundamental property of allowing non-directed investigations as well as procedures where there is a target objective. For the latter case, HRMS and QqQ are both frequently applicable in the analysis of veterinary drug residues [22].

Authors reported that the use of HRMS screening methods has an advantage over the traditional methods of MS since it increases the scope for monitoring food from unexpected veterinary drug residues.

Anumol et al. [35] compared UHPLC-QqQ vs. UHPLC-QToF and analyzed veterinary drug residues in animal tissues with different extraction procedures. They concluded that based on the interference of the analyte and the matrix, the detection of QqQ vs. QToF showed similar mixed performance advantages, while the advantage of QToF lies in the larger analysis range and non-target data collection. The results showed that for 80% of the 127 veterinary drugs, both extraction methods provided global mean recoveries ranging from 70% to 120% in bovine tissues. In conclusion, they demonstrated that the latest EMR-L system and UHPLC-QToF study, both separately and in combination, are viable alternatives for analyzing common veterinary drugs.

Kaufmann [113] reported his perspective on the HRMS technique. From his point of view, the technology currently used in bioanalytical applications is still considered a screening technique or a research tool. Despite its continuing discussion in a scientific setting, it has not been widely used in a routine laboratory setting, and there is still an unwillingness to use HRMS for quantitative measurements in a regulatory setting. Furthermore, he reviewed the power of three mature, commercially available instrument options: the QTOF, Orbitrap, and Q-Orbitrap configurations capabilities. The power of these configurations as alternatives to the QqQ analyzer is discussed based on practical examples derived from his own workplace. The incorporation of the quadrupole in the routine is used like a wide-pass filter to eliminate very light and very heavy ions or to allow the passage of only a specific mass range of interest. Although in none of these options, the quadrupole is used as the primary analyzer, he concluded that it is still to be shown that HRMS can produce equally reliable, accurate, and precise results as QqQ [113].

In particular, LC was used in conjunction with a triple quadrupole (QqQ) mass spectrometer with relatively long dwell times (>100 ms). The number of compounds that could be detected in a single run was limited due to the LC system’s large dwell durations and low resolution. In the last ten years, high-resolution LC has achieved sub-2 µm stationary phase particles and rapid scanning (<10 ms) [35,72,96,101,106]. With the QqQ instrument, it is easy to detect more compounds in one run [9]. Therefore, effective sample preparation is very important to avoid high back pressure and/or column clogging [9].

In recent years, the Orbitrap system has gained popularity due to its high resolving power, high dynamic range, and, as a result, better mass accuracy compared to ToF systems [88]. Moreover, during the development of a multiresidues method, laboratories waste a considerable amount of time during the validation process. Routinely, the detection of numerous compounds is low, so screening methods are increasingly used for rapid analysis of samples at lower costs (decrease in the total number of samples needed to be valid). One of their key benefits is that they can make quick decisions and have a relatively easy and flexible extraction method [9].

Turnipseed et al. [102] studied the application of a screening technique for fish species that have been treated with various classes of veterinary drugs, including complicated analytes such as aminopenicillins, dyes and avermectins. Furthermore, the procedure was used on samples of imported fish. Prior to LC-HRMS screening, the fish samples were determined using a validated LC-MS/MS triple quadrupole method (Q-Orbitrap). The HRMS screening methodology allowed detection and identified new analytes, such as ofloxacin in corvine and 2-amino mebendazole in eel. The paper shows how this HRMS technology can be used to study fish and crustaceans for routine use. Pugajeva et al. [101] reported an analytical method for screening and quantification of 164 residues and metabolites of pharmacologically active substances through the UHPLC system combined with the Q-Orbitrap HRMS mass spectrometer. A recovery range of 70 to 120% was obtained. The method’s success was demonstrated by the identification of compounds above the CCβ levels, suggesting that it is a good screening technique for routine research.

### 5.2. Non-Chromatographic Methods

Another approach used for the detection of antibiotics in food, either qualitative or semi-quantitative form, is the use of immunological methods such as enzyme-linked immune sorbent assay (ELISA), fluorescence immunoassay, radioimmunoassay, colloidal gold immunoassay, and chemiluminescence immunoassay. Recently, new immunoassays for detecting antibiotic residues have been developed, including surface plasmon resonance immune technology, immune chip technology, immunosensors, and others [114]. One of the most promising for the multi-analysis of antibiotics is the biochip assay. It is made up of a set of microarrays arranged on a solid substrate, allowing many experiments to be performed at the same time. The biochip assay is focused on the precise recognition of the analyte’s target bond in biological receptors or molecules in an orderly sample, allowing the analyte to be identified quantitatively or semi-quantitatively. Multiple targets can be analyzed in a single test by modifying assay formats and using wide specific antibodies. With the advancement of science, it is expected that more methods will be used in immunoassay [114].

In a recent review article, Majdinasab et al. [115] focused on new achievements in the development of biosensors for the detection of antibiotics in food. Various types of chemical sensors are discussed, including enzymes, antibodies and nanobodies, aptamers, DNAzymes, MIPs, and antibiotic detection cells. Different groups of nanostructures, including carbon-based nanomaterials, metallic nanomaterials, quantum dots (QDs), upward converting nanoparticles (UCNPs) and magnetic nanoparticles (MNPs) integrated into the antibiotic biosensor detection platform and detection strategies, including optical methods (colorimetry, fluorescent, chemiluminescence- CL) and surface plasmon resonance (SPR), electrochemical biosensors and sensitive mass-based biosensors for monitoring antibiotics are discussed. Different classes of nanostructures, such as carbon-based nanomaterials, quantum dots (QDs), metallic nanomaterials and metallic nanomaterials upward converting nanoparticles (UCNPs) and magnetic nanoparticles (MNPs), are addressed as they are incorporated into the antibiotic biosensor detection network and detection techniques, such as surface plasmon resonance (SPR) and optical methods (colorimetry, fluorescent, chemiluminescence-CL), sensitive mass-based biosensors and electrochemical biosensors for monitoring antibiotics. The authors summarized the advantages and disadvantages of each type of biosensor. Fluorescent biosensors, for example, can be an excellent option for quantitative and semiquantitative detection. Through the use of nanomaterials such as UCNPs, its sensitivity has recently increased to pg/mL. The disadvantage is that a fluorophotometer is needed to read the result. Another option is the colorimetric biosensors with adequate sensitivity and with quick results. In this situation, the use of DNAzymes and signal amplification will greatly boost sensitivity, but the increased test time is a disadvantage. Regarding biochemical sensors, it is the opinion of several authors that improvements in the performance of this type of biosensors are necessary. The development of good electrode material, metal nanoparticles, metal oxide, and carbon nanostructures for the construction of electrochemical biosensors can result in high sensitivity and speedy performance. Other types of biosensors benefit greatly from the use of nanomaterials as chemical alternatives. Nanomaterials can improve optical and magnetic properties in optical biosensors, allowing for greater sensitivity and precision in detection [115]. Because of their excellent stability, long duration, ease of synthesis, low manufacturing costs, and in vitro development, MIPs show promise. The simultaneous identification of different classes of antibiotics is being developed in the biosensor array based on the mobilization of numerous bioreceptors to recognize different antibiotics at the same time, which can decrease analysis time and cost. Yue et al. [116] reviewed recent developments and uses of aptamer-based sensors for the detection of aminoglycoside antibiotics. They selected nine detection methods for aminoglycosides: optical fluorescence, colorimetric, CL, surface-enhanced Raman scattering, electrochemical impedimetric, voltammetric, potentiometric, electrochemiluminescence and photoelectrochemical. The authors concluded that each aptasensor has its own distinct properties and that selecting any tool to detect aminoglycosides is dependent on the various conditions and goals. As a result, there are still some obstacles to overcome before aptasensors can be used to detect aminoglycosides. To begin, the aptamer for aminoglycosides is chosen based on environmental factors such as temperature, pH and ionic strength. The aptamer, on the other hand, is used for the fabrication of the aptasensor in measuring settings, which can vary from selection conditions and affect the specificity and affinity of aptamers.

## 6. Effect of Food Processing on the Residues of Antibiotics Found in Animal Products

When veterinary medicines are administered to animals, residues can remain in meat, milk or eggs if proper precautions are not followed. The application of MRL in raw foods does not consider the changes that occur during the processing of these foods. Since the largest foods of animal provenience are usually eaten after preparation, it is critical to consider the effects of various heat treatments on residues when evaluating human exposure, determining MRL, and assessing toxicity [8,117].

Regarding research on the effect of heat procedures on antibiotic residues, usually, they show their results as a percentage of degradation after treatment. Based on the available literature, it can be inferred that the respective heat treatment reduces the concentration of antibiotic residues or bioactivity in the food product. As a result, the values published in the literature vary widely depending on the type of treatment utilized, pH, temperature and matrix [117]. Food cooking can be done in a variety of ways, including boiling, scalding, steaming, baking, roasting, frying, microwave cooking, grilling, barbecuing, smoking, sous vide and confit. These procedures involve the application of heat at different temperatures and times for food preparation. The percentage of β-lactams residues in food that degrade after cooking ranges from 0.1% to 100%.

It is reported that the stability of β-lactam under heating is very low, a consequence of the high ring strain of the small β-lactone ring, which contributes to their hydrolysis.

The molecules of cephalexin and cefuroxime are unstable in biological matrices, including at moderate temperatures of 60–80 °C; they are more susceptible to heat than other β-lactam antibiotics. In the case of meat, long-term roasting resulted in a high degree of ampicillin degradation. For milk and water, when a classic sterilization procedure is applied (120 °C for 15–20 min), β-lactams antibiotics are significantly reduced [117]. Canton et al. [118] investigated how cooking affects the stability of veterinary drug residues in chicken eggs.

For the study of the degradation of the AMX, eggs can be prepared at different times and in different ways (methods), namely, making omelets, microwaving, heating, and boiling. The major reduction in egg residues was proportional to the cooking time when measuring the stability of AMX residues by boiling. After microwaving or making an omelet, there was a substantial loss of water. The AMX residue in eggs was unstable and was significantly reduced during all cooking processes. The amount of AMX residue reduction is proportional to the time each cooking process takes to cook (microwaving, cooking, boiling and omelete making). During the microwaving and omelet-making processes, the most residue was removed. According to reports, penicillin G of AMX in milk is degraded by about 20%, cephalexin by about 27%, and cefuroxime by about 35%.

In the past, meat has been shown to degrade penicillin. According to O’Brien et al. [119], the degradation of ampicillin after roasting bovine tissue is highly variable and appears to be dependent on the temperature reached while cooking as well as the cooking time. It is not known to the author if, until now, there is a conclusive and effective study about the effect of degradation of this class of antibiotics in food.

Research studies demonstrate that the kind of food matrix and cooking process affect TC deterioration in chickens and pigs. The stability of TC, tetracycline, OTC, CTC, and DC in the chicken thigh and breast samples after boiling, roasting, and microwaving was studied using varied temperatures and periods. Tetracycline degradation percentages vary from 2% to 100% when subjected to heat treatments [117].

Cooking time increases TC loss, with DC being the most heat-stable and OTC being the least. For example, to ensure 90% TC destruction in chicken meat, microwave (2450 MHz), boil (100 °C), and roast (180 °C) it for 24, 53, and 102 min, respectively. This suggests that standard cooking methods will not be sufficient to destroy these antibiotics [120].

Time and temperature are the two most important elements in reducing antibiotic residue during cooking. Microwaved, roasted and cooked chicken resulted in 74%, 48% and 35% OTC losses, respectively. There was a decrease in the OTC in the frying process. This effect can be attributed to water loss in chicken meat [121]. Shaltout et al. [122] investigated the impact of microwave and boiling treatments on OTC residue reduction in chicken muscles. The percent of reduction waste was 81.48% and 77.93%, respectively. Grilling and roasting, according to research, are the most efficient and least effective cooking procedures for reducing OTC and DC concentrations in chicken, respectively. Only one investigation on the stability of OTC in shrimp samples was reported in the last decade in the case of fish. Kleechaya et al. [123] studied the degradation of OTC in black tiger prawns. The obtained results were a reduction of residual OTC by 30–60% by boiling, baking and frying, while in the shell, OTC was reduced by 20% in each cooking method. The study of the degradation of TC’s in their respective epimers, 4-epi-TC, 4-epi-OTC, 4-epi-CTC, 4-epi-DC and anhydro-TC’s is extremely relevant for risk assessment in the consumer. TCs degrade in various ways depending on the pH of the medium. Thus far, there has been little research into the toxicity of TC breakdown products. Anhydrotetracyclines are considered hazardous, causing reversible kidney damage. However, it is unknown if hazardous degradation products will be generated in considerable quantities during ordinary household cooking processes due to a paucity of studies on the characteristics of TC degradation products during diverse processing circumstances [117].

The thermal degradation percentages of macrolides ranged between 0% and 93%. [117]. Erythromycin is the most heat-sensitive compound of the macrolide family. Milk heat treatment studies for 20 min at 120 °C showed that the residual amount of erythromycin was reduced by more than 90%, while the residual value of other macrolides was much lower [117]. Reduction of tilmicosin of 37%, 46% and 41% in boiling, frying, and microwaving cooking methods, respectively, was related by Hussein et al. [121]. Salaramoli et al. [124] used HPLC to determine the amount of tylosin in raw and cooked samples. The study’s findings revealed that when cooked and uncooked chicken meatballs were compared, the cooked samples had a significant reduction in tylosin amounts, both by microwave heating and boiling.

Chloramphenicol, florfenicol, and thiamphenicol are examples of broad-spectrum antibiotics in the amphenicol group. It was demonstrated that the bioactivity of chloramphenicol in beef after roasting for 2 h decreased by 70%. Its degradation in beef is almost 5 times higher than in water [117]. These conclusions were reported by Franje et al. [125] and Clarke et al. [126]. Both reported that the greater degradation of chloramphenicol, which is lipophilic, could be due to the meat’s low water-binding ability after heating. [117]. The class of quinolones is not much affected by processing methods. Oxalinic acid and flumequine in salmon, enrofloxacin and ciprofloxacin in Latin fish were highly stable during heating [127]. Boiling, roasting, and frying, on the other hand, minimize the content of oxolinic acid in shrimp by 20% to 30% [128]. Heating methods such as frying, boiling, grilling, microwave cooking, and roasting all appear to affect quinolone residues in meat samples. Boiling and microwaving reduced enrofloxacin levels in the uncooked thigh and chicken breast muscles, whereas oven-roasting and grilling raised them [120].

Hasanen et al. [129] studied ciprofloxacin residues in chicken and turkey carcasses. They concluded that ciprofloxacin residues are heat-stable and are not degraded by any cooking method, except microwaves (800 W) for 15–20 min in muscles and 3–5 min in the liver and kidney, also freezing for one month at −20 °C can degrade ciprofloxacin and its metabolites to levels below the permitted limits, but not below detectable levels. Roca et al. [130] related quinolones’ stability by heating milk. Quinolones are very resistant to heat treatments, with maximum concentration losses of 13 percent for ciprofloxacin and 12 percent for norfloxacin at 120 °C for 20 min. Quinolones’ high stability poses a risk to human health because antibiotic residues will remain in milk after heat treatment and thus enter the dairy industry and consumers [130]. A study by Ismail-Fitry et al. [131], in relation to the effect of deep-frying at different temperatures and times on sulfonamide (SA) residues in chicken meatballs, concluded that frying chicken meat-balls at 180 °C for 6 min results in better appearance and quality status of meat for consumption as well as a reduction of SA residues. Deep-frying could aid in the reduction of SA residues in chicken meatballs, with maximum reductions of 38, 28, 41, and 28% obtained for sulfadiazine (SDZ), sulfamethazine (SMZ), sulfamethoxazole (SMX), and sulfaquinoxaline (SQX) at the maximum frying time and temperature, respectively. Ismail-Fitry et al. [131] concluded that the increasing order of degradation of sulfonamides was deep-frying, boiling and microwaving, while the SDZ was the most heat-labile SA. Javadi et al. [132] used a microbial inhibition approach to reduce the concentration of sulfadiazine and trimethoprim residue in broiler edible tissues after various cooking processes. The microwave method is similar to the one that causes the greatest reduction in SDZ and trimethoprim residues in cooked muscle samples.

Zhao et al. [133] studied the degradation kinetics of six kinds of SA, SDZ, SMX, sulfasalazine, SMZ, SMX and sulfadimethoxine in eggs at simulated cooking temperatures. SDZ and sulfadimethoxine had the lower and biggest or ample half-life time, respectively. The LC-MS/MS methodology was used by Roca et al. [134] to investigate the kinetics degradation of eight types of SA, sulfadimethoxine, sultathiazole, sulfapyridine, sulfacloropiridazine, sulphaquinoxaline, SDZ, sulfamerazine and SMZ when skimmed milk is heated at 60, 70, 80, 80, 90 and 100 °C. The results obtained show that sulphonamides are very stable molecules that can resist even the most common heat treatments performed in the dairy industry without degrading significantly. The degradation of sulfamerazine, SMZ, SDZ, and SQX in milk was explained by the slow reaction rate at low temperatures and the quick increase at high temperatures. The high collision energy between molecules was sufficient to disrupt the pre-existing connection, resulting in a higher degree of degradation [134]. The collision level of sulfadimethoxine and sulfathiazole, on the other hand, was low, indicating that the reaction rate and degradation rate were both low [117].

## 7. Conclusions and Future Perspectives

The development in the instrumentation of LC-MS made possible the analysis of multiresidue multiclass veterinary residues in a single analysis run. HRMS has grown in popularity for bioanalytical applications because it is very effective at both detection and quantification. The use of LC-ToF-MS and Orbitrap seems to be a trend for the detection of compounds through full-scan that allows fast runs and good reproducibility of results. The implementation of screening methods using HRMS technology is increasing, replacing LC coupled to quadrupole-based tandem mass spectrometry, thus reducing response time and analysis costs. Nonetheless, despite significant technological advancements at the instrumental level in terms of detector selectivity and specificity, the sample preparation phase remains critical in the entire analytical process. The complexity of the nature of the sample requires efficient extraction and purification that allows reaching the required low residual levels and ensures the reduction of maintenance need of the equipment. This goal becomes even more demanding in the analysis of multiresidues multiclass. Typically, these methodologies include a sample cleaning and/or pre-concentration step appropriate for the number of compounds and different classes included in the analytical procedure. The SPE technique is the most used in the area of antimicrobial residue analysis. Related to the development of these techniques is the concept of green analytical chemistry, where the decrease in the sample size, reduction in the volume of solvents in the extraction step, especially of organic solvents, automation and low costs have been increasingly optimized.

The effect of food processing on the residues of antibiotics found in animal origin foodstuffs was also addressed in this work. It was discovered that cooking time and temperature are the deciding factors affecting antibiotic reduction in foods. Tetracyclines are highly degraded by frying, roasting, and boiling. However, research gaps have been detected regarding the compounds resulting from this degradation. There are also no concise studies concerning the effect of food processing on β-lactams and macrolides, as these two classes of antibiotics are widely used in the veterinary field.

Several parameters related to matrix composition, such as nutrients like fats or sugars, pH, additive addition, and cooking methods, all influence antibiotic degradation. However, there is still a lack of information regarding the extension of their effect. For example, it is known that thermal processing leads to a decrease in the levels of antibiotic residues, but little is known about the characterization of their by-products.

One of the major future challenges in the frame of food safety is to understand the factors and degradation mechanisms of veterinary drugs during food processing, and the characterization of the resulting new compounds, in terms of their chemistry, biological actions and toxicity.

## Figures and Tables

**Table 1:** Summary of the Appendix A (List of non-compliant results: targeted sampling) of the technical report of the monitoring data collected in 2018 on the presence of residues of veterinary medicinal products and certain substances in live animals and animal products in the European Union. European Food Safety Authority. Approved: 20 December 2019.

Class	Substance	Aquaculture	Bovine	Eggs	Honey	Milk	Pig	Poultry	Rabbits	Sheep/goats	Eggs
Sulfonamides	Sulfadimethoxine		√	√			√		√	√	√
	Sulfadimidine		√				√				
	Sulfamethoxypyridazine		√								
	Sulfacetamide				√						
	Sulfachlorpyrazine				√						
	Sulfadiazine			√	√		√			√	√
	Sulfadimidine				√						
	Sulfamethazin				√						
	Sulfathiazole				√						
Tetracyclines	Oxytetracycline	√	√		√	√	√	√		√	
	Epi-Oxytetracycline		√								
	Chlortetracyclin		√								
	Doxycycline			√			√	√			√
Penicilines	Amoxycillin		√			√	√				
	Benzylpenicillin (Penicillin G)		√				√				
	Ampicillin									√	
	Cloxacillin					√					
Macrolides	Gamithromycin		√								
	Spiramycine		√								
	Neospiramycin										
	Tilmicosine		√	√			√			√	√
	Tulathromycin		√							√	
	Erythromycin						√				
Quinolonas, including fluoroquinolones	Enrofloxacin		√	√			√	√	√	√	√
	Oxolinic Acid			√							
	Ciprofloxacin								√	√	
	Oxolinic Acid										√
	Danofloxacin		√				√				
	Sarafloxacin						√				
	Flumequine	√						√			
Aminoglycodides	Dihydrostreptomycin		√		√		√			√	
	Neomycin		√								
	Streptomycin				√						
	Aminosidin (Paromycin, Paromomycin)					√					
Phenicols	Florfenicol		√								
Lincosamides	Lincomycine		√								
Diaminopyrimidinas	Trimethoprim	√		√					√		√

**Table 2:** Summary of the most common techniques currently available for clean-up and extraction of antibiotic residues in foodstuffs. The table is completed with the application of the methodology in real samples, relative levels of contamination, recovery percentages and conclusions of the study. The table was configured in chronological order for better visualization of the methodologies trends to date.

Type of Sample	Class of Antibiotic Residues Analyzed	Extraction Methods	Extraction Conditions	Clean-Up	# Samples	Sampling Period	Levels of Contamination	Recoveries	Conclusions of the Study	Ref.
Milk	BZM, AT	QuEChERS d-SPE	10 g + 10 mL AcN 0.1% NH3 + 5 g MgSO4: NaCl (4:1, *w/w*). Shake for 1 min, centrifuge at 5000 rpm for 5 min.	Transfer 1 mL of the supernatant to a 2 mL eppendorf containing MgSO4 (150 mg), C18 (50 mg) and PSA (50 mg). Vortex for 1 min and centrifuge at 3000 rpm for 2 min. Evaporate an aliquot of the supernatant (500 µL) to dryness and reconstitute in acetonitrile:water (5:95).		2013	10–100 µg/Kg	75–100%	The application of a modified version of the QuEChERS method assured a fast sample treatment with low MLOQ (<3 µg/Kg), allowing the determination of these compounds at concentration levels considerably lower than the established EU MRL values.	[70]
Poultry muscle	MA	SPE	5 g + 20 mL EDTA-McIlvaine’s buffer pH 3.5. Mix and centrifuge at 5000 rpm for 10 min. Centrifuge again twice more with 20 + 10 mL EDTA McIlvaine’s buffer, respectively.	Conditioning the Oasis HLB cartridges (500 mg) with 5 mL of MeOH and 5 mL of water. Pass the extract. Wash the columns with 5 mL of water and elute with 5 mL of MeOH. Evaporate the extract to dryness. Re-dissolve with 1 mL of the 0.05% TFA (pH 2.5)/ACN (70:30, *v/v*) mixture.	30	2013	200 µg/Kg	85–120%	The spiramycin and tylosin were not detected in the analyzed samples. Only tilmicosin was found in two broiler chickens at 38 µg/Kg (supermarket) and 65 µg/Kg (wet market) with RSDs (%) (n = 2) of 0.2% and 0.4%, respectively. The tilmicosin did not exceed the maximum permitted limit (MRL) of drug residues in chicken muscle, which was 150 µg/Kg according to EU Regulation and 100 µg/Kg according to the Malaysian Food Act.	[60]
Milk and muscle	MA, LS	LLE	2 mL of milk + 4.0 mL of ACN, divided into 3 aliquots (2.0 mL + 1.0 mL+ 1.0 mL). Shake for 20 min and centrifuge for 5 min at approximately 3000 g, at 5 °C. 2 g of muscle + 10.0 mL of ACN. Homogenize with a mechanical mixer. Shake for 20 min and centrifuge for 5 min, at approximately 3000 g, at 5 °C.			2015	50–100 µg/Kg	60–73% (Milk) 60–107% (Muscle)	After LLE, followed by centrifugation, the obtained organic extract was very limpid and adequate to be directly injected into the LC–MS/MS system. The sample preparation method has shown high efficiency in extracting the target compounds. Fast and environmentallyfriendly once it generates fewer solvent residues than other methods that use SPE.	[31]
Milk	PN	LLE	2.0 mL + ACN (2 mL + 1 mL + 1 mL) with manual mixing between each addition. Mix 15 min. Centrifuge 5 min at 4000 g, at low temperature (5 °C).	Transfer the supernatant to a 50 mL tube with 100 mg of C18 bulk. Shake and centrifuge for 5 min at 4000 g. Take the supernatant in a freezer (−17 °C) for 20 min. Centrifuge (10 min at 4000 g, 0 °C). Transfer the supernatant and evaporate to approximately 500 µL	247	2015	0.10, 0.25xMRL	58–108%	From a total of 247 samples, two samples had positive residues; one sample contained cloxacillin (107 ng/L), and another contained cefapirin. The sample preparation method was not complex and is environmentally friendly when compared with reports for β-lactam analysis in food samples, which generally require the use of SPE associated with additional clean-up steps. Validation data were satisfactory, showing agreement with the criteria proposed by Commission Decision 2002/675/EC.	[57]
Butter, egg and milk powder	QN, TC, CP, MA, SA, BZM, NMZ, CCD, NSAIDs, AM, CDC, Antiepileptic drugs, Fibrates and Others (115)	LLE	1 g + 2 mL of H_2_O containing 0.1% formic acid (*v/v*) and 0.1% EDTA (*w/v*). Mix and add 2 mL ACN + 2 mL MeOH. Mix for 30 s. Put the samples in an ultrasonic bath at 600 C for 20 min. Centrifuge at 4000 rpm for 10 min.	Transfer the supernatant and precipitate at 23 °C for 12 h. Centrifuge again. Defatting with 5 mL of hexane. Vortex for 1 min. Centrifuge at 4000 rpm for 10 min. Discard the n-hexane layer. Evaporate to dryness. Re-dissolution in 1 mL of MeOH/aqueous formic acid solution, 0.05% (25:75 *v/v*)	108		1–200 μg/Kg	Butter: 50–120% Fish tissues: 50–9% Eggs: 50–80% (66 compounds) Milk: 50–80% (78 compounds)	Oxfendazole and albendazole sulfone were detected in two milk powder samples at 1 μg/Kg and 0.54 μg/Kg, respectively. These concentrations, although they are higher than LOQ, are very low and far below the MRL established for these benzimidazoles in milk (10 μg/Kg and 100 μg/Kg). Several quinolones were detected in milk (ciprofloxacin at 7.3 μg/Kg and norfloxacin at 2.2 μg/Kg) and fish tissue samples (flumequine at 4.6 mg/kg and enrofloxacin at 4.8 mg/kg). Thiabendazole was detected and quantified at 9.7 mg/kg in one butter sample, and, finally, caffeine was present in six milk powder samples at concentrations ranging from 2.4 to 32 mg/kg. No compound detected and quantitated would exceed the MRL established in Commission Regulation 37/2010/EC.	[47]
Eggs	Amoxicillin and metabolites	LLE	5 g + ACN. Mix for 15 min. Centrifuge at 3500 g at 4 °C for 10 min. Repeat the extraction. Adjust the volume to 1 mL with ammonium acetate (3.89 mol/L, pH 6.74). Add 20 mL of dichloromethane. Shake and centrifuge at 3500 g, 4 °C for 5 min. Transfer 1 mL of the supernatant and evaporate to dryness. Reconstitute with 2 mL of 2% acetonitrile in water.		40	2015	0.1–0.6 µg/Kg	80.15–98.23%	Compared with previous publications, this method presented fewer interferences and higher recovery rates (≥80%). The major advantage of this protocol is its simplicity and short analysis time. One sample was contaminated with 4.53 µg/Kg amoxicillin (<MRL), while amoxicilloic acid, diketopiperazine-20,50-dione and ampicillin were not found in any samples.	[58]
Muscle, liver and kidney	AG	SLE, SPE	5.0 g + 10 mL of 5% TCA (*w/v*). Mix and centrifuge at 5 °C, 8000 rpm. Repeat with 10 mL of 5% TCA. Add 5 mL of 0.2 mol/L HFBA + 5 mL n-hexane. Shake, and centrifuge at 3600 rpm for 30 min. Remove the n-hexane phase.	Conditioning the HLB columns with 3 mL of MeOH + 3 mL of water + 3 mL of 0.2 mol/L HFBA. Transfer 5 mL of the extract. Adjust the collected eluent to pH 8.5 ± 0.2 with 100 g/L NaOH + 0.2 mol/L HCl. Dry the columns. Condition other HLBs with 3 mL of MeOH + 3 mL of water + 3 mL of 0.2 mol/L of HFBA + 3 mL of aqueous NaOH pH 8.5. Transfer the effluent to pH 8.5 ± 0.2 in the column. Connect the two HLB cartridges with vacuum joints. Wash the two tandem cartridges with 5 mL of water and dry. Elute with 6 mL ACN/0.15 mol/L HFBA (4: 1, *v/v*). Evaporate to 0.3 mL. Reconstitute with 1 mL 20 mmol/L HFBA.		2016	0.5, 1, 1.5 x MRL	Muscle: 47–64/7–85% Liver: 5–68/70–85% Kidney: 56–70/70–85%	The method showed good sensitivity, and the performance characteristics comply with EU recommendations. This method should be an efficient approach for multiresidue analysis of AGs in animal tissues.	[71]
Bovine muscle	AG, PN, MA, SA, AT, CD, NSAIDs, Pharmaceuticals, Rifaximin, Baquiloprim, Trimethoprim, Chlorpromazine, 6-phenyl-2-thiouracil, Bromhexine (76)	SLE, SPE	5 g + 10 mL of ACN. Centrifuge. Decant the supernatant. Evaporate to 1.0 mL. Add 20 mL of the 0.4 mM EDTA solution + 1% NaCl (*w/v*) + 2% TCA (*w/v*). Shake for 60 min on a mechanical stirrer. Centrifuge at 4000 rpm for 5 min. Decant the supernatant, and adjust the extract to pH 6.5 by adding approximately 6 drops of 30% NaOH (*w/v*).	Conditioning OASIS HLB Cartridge (200 mg, 6 mL), with 6 mL MeOH + 6 mL H_2_O. Pass the sample through the columns and dry it in a vacuum for 15 min. Elute with 2 × 0.5 mL of 10% (*v/v*) aqueous formic acid + 3 × 1 mL of ACN. The eluate is collected and combined with 1 mL ACN extract.	10	2016	5–1000 µg/kg	37.4% (bromhexine) to 106% (kanamycin).	No positive results were found in any of the analyzed samples. The low recoveries of some compounds are considered acceptable since they were reproducible.	[72]
Animal tissue (muscle), honey, milk	AG	LLE, SPE	Honey: 2 g + 10 mL of solution A (50 mmol/L potassium phosphate). Shake for 5 min. Adjust the pH = 7.0 with ammonia solution (28.0–30.0 %). Animal tissue and milk: 2 g + 5 mL of solution B (10 mmol/L potassium phosphate monobasic (KH2PO4) + 0.4 mmol/L EDTA-Na2 + 2% TCA). Vortex for 5 min. Centrifuge at 3200 rpm, 4 °C, 5 min. Transfer the supernatant, add 5 mL of solution B and repeat the extraction process. Dilute the supernatant with 1:1 solution A. Adjust the pH = 7.0 with ammonia solution (28.0–30.0%).	Conditioning SupelMIP SPE-AGs cartridges (3 mL/50 mg) with 1 mL MeOH + 1 mL 50 mM K_3_PO_4_ (pH 7). Pass the extract. Wash the columns with 3 mL of water + 1 mL of 0.1% ammonia solution at 0.5 mL/min (the ammonia washing step is not carried out for milk and pork samples). Apply strong vacuum, 5 min. After washing step with 0.1% ammonia. Wash with 1 mL of acetonitrile-water (40 + 60, *v/v*) + 1 mL of methanol-dichloromethane (50 + 50, *v/v*). Elute with 1 mL of 1% formic acid in MeOH-water (80 + 20, *v/v*).		2016	20–300 µg/kg	78.2–94.8%	The MIP proved to be a powerful and selective material for the cleanup of extract from complicated food matrixes.	[73]
Muscle, urine	PN, CP, SA, TC, MA, LS, QN, AM, DP (29)	SPE	Muscle: 1 g + 5 mL McIlvaine buffer (pH 4.0) + TCA (100 mL, 20% *w/v*). Vortex. Centrifuge at 2500 g, 4 °C, 10 min. Transfer the supernatant to a new centrifuge tube. Degrease with 2 × 3mL of n-hexane. Discard the n-hexane layer at each centrifugation (2500g, 5 min). Urine: 5 mL centrifugue at 2500g, 4 °C to 5 min.	Muscle: Conditioning the Oasis HLB cartridges with 3 mL of MeOH + 3 mL of water under vacuum. Transfer the extract to the cartridge. Wash the cartridge with 2–3 mL of MeOH: water (5:95 *v/v*). Elute with 5 mL of MeOH and collect. Evaporate the eluate. Reconstitute in 200 mL of MeOH: water (10:90 *v/v*). Urine: Conditioning the Oasis HLB cartridges (3 mL, 60 mg) with 3 mL of MeOH + 3 mL of 0.5 M HCl + 3 mL water, under vacuum. Transfer 5 mL of urine to the cartridges. Wash with 3 mL of water + 3 mL of MEOH: water (20:80, *v/v*). Elute with 5 mL of MEOH and collect. Evaporate the eluate. Reconstitute in 200 mL of MeOH: water (10:90 *v/v*).	86 (43 urine; 43 muscle)	2017	Muscle: 1–10 ng/mL Urine: 0.5 ng/mL	Muscle and urine: 90–106%	Doxycycline was one of the antibiotics most frequently found in urine (in 37 samples, at a maximum of 339.45 µg m/L). In the muscle tissues, doxycycline was found 15 times, and the maximum recorded was 21.05 µg/kg. No strict correlation was found between the two matrices, but generally, when the concentration in the urine was very high, the analyte was detected in the paired muscle sample.	[44]
Meat, Milk, Egg, Fish	AM, AT, BZM, PN, CCD, IO, MA, NAIDS’s, QN, SA, TC, TQ (100)	d-SPE, LLE	2 g tissue (muscle, liver, or kidney) + 10 mL mixture of ACN and water (4:1, *v/v*) containing 2 mM ammonium formate. 1 g of milk + 2 mL ACN the resulting mixture, was put into an ultra-centrifugal filter (cut-off membrane at 3 kDa, 4 mL) and centrifuged. 2 g of egg and fish + 3 mL of ACN + 1 mL of water contains 2 mM ammonium formate. Centrifuge 5000× *g* at 4 °C, 5 min. The supernatant (2 mL) was purified by centrifugation (5000× *g*, 4 °C, 60 min) in an ultra-centrifugal filter (cut-off membrane at 3 kDa) + 2 mL of hexane. Vortex shake and re-centrifuged.	The tissue in solvent mixture was homogenized, shaken for 5 min, and centrifuged. 10 mL hexane + 250 mg octadecyl carbon chain (C18)-bonded silica.Vortex shake for 30 s. The mixture was centrifuged for 5 min at 10,000 rpm. Discharged the n-hexane. The extract was evaporated under nitrogen until near dryness.		2016	5, 10, 20, 50, 100 µg/kg	63 to 122%	The simple pretreatment and rapid detection method significantly reduced the time (2–3 h), human resources, and hazardous organic solvent requirements compared with conventional methods. Good ccα’s and good recoveries in general for the three matrices.	[74]
Meat (pork, chicken, fish tissues) and Eggs	SA, QN	UAE, SPE	2 g + 10 mL of 0.1% formic acid in 90:10 ACN/water (*v/v*). The mixture was subjected to UAE for 10 min at 20 °C and then centrifuged at 8000 rpm for 15 min at 4 °C. Remove the supernatant.	Pass a 2 mL aliquot of the supernatant in Oasis PRMiME HLB 3 cc Vac Cartridge, 60 mg. Collect the eluate and evaporate to dryness under mild ultra-high purity nitrogen gas at 35 ° C. The resulting residues were reconstituted in 1 mL of initial mobile phase (10 % MeOH).	151	2017	2.5, 5.0 and 10.0 μg/kg	Pork: 61.9–85.1%/95.4–121.6% Chicken: 51.9–58.6%/95.4–121.6% Fish: 42.2–51.1%/106.7–116.1% Eggs: 57.1–73.9%/108.7–122.4%	Sulfacetamide at 1.2 μg/kg in one pork sample, sulfaquinoxaline at 3.3 μg/kg in another pork sample, and sarafloxacin at 0.98 μg/kg in one egg were detected. These concentrations are very low and far below the MRL.	[68]
Fish muscle	TC (5)	QuEChERS	1 g + 2.2 mL of EDTA-McIlvaine buffer + 5.0 mL for ACN + 1.25 g of (NH4)_2_SO_4_. Shake for 1 min, centrifuge at 9000 rpm for 5 min.	Transfer 5.5 mL of the supernatant to polypropylene tubes with 50 mg of C18. Vortex for 1 min, centrifuge for 5 min at 5000 rpm. Transfer 5 mL of the supernatant and bring to dryness. Reconstitute with 500 µL of H2O: MeOH (95: 5, *v/v*)	10	2018	5, 20, 100 μg/kg	80–105%	Only traces of oxitetracycline were found in one salmon sample, obtaining a concentration of 5.6 ± 2.2 µg/kg. LOD between 0.6–1.3 μg/kg and LOQ between 1.7–4.0 μg/kg. Good precision.	[42]
Muscle, kidney, liver	TU, NMZ, LS, AT, TC, PN, QN, BA, CP, SA, MA, AM, CDC, TQ, Flukicide, NSAID, Progestin	SPE	2 g + 2 mL 0.1 M EDTA. Shake and centrifugate. Transfer the supernatant. Add 8 mL of cold ACN w/2% FA and 2% DMSO to the sample residue left in the first tube. Shake and centrifuge. Decant supernatant was placed in the second tube and combined with the previous extract. Vortex the mixed extract in the second tube and centrifuge.	Transfer 5 mL of supernatant to EMR-Lipid 6 mL cartridge. Add 1.25 mL 20:80 water/ACN into EMR-Lipid 6 mL cartridge. Collected eluent and combined 0.5 mL of sample eluent with 0.3 mL of water.		2018	20/4 ng/g (G1/G2)	60–120% (for 90% of tested veterinary drugs)	EMR-Lipid cartridge cleanup provides high matrix co-extractive removal and reduces the matrix ion suppression on the target analytes. The quantitative analysis results showed that >90% of tested veterinary drugs provided acceptable recoveries, and >95% of compounds gave excellent reproducibility in all five meat matrices.	[51]
Poultry muscle	TT, BA, QNX, PN, LS, MA, NMZ, BZM, NSAIDs, QN, SA, TC, CCD, AT, CDC, PP, Pesticides and others (140)	SLE, d-SPE	5g + 0.2 mol/L ethylenediamine tetraacetic disodium salt (EDTA–Na2) + 12.5 mL of ACN:ethanol (80:20, *v/v*). Defatting twice with 10.0 mL of n-hexane and centrifuge. Transfer ACN–ethanol aqueous phase to another tube with another 10 mL of ACN:ethanol (80:20, *v/v*). Remove the supernatant, transfer it to another tube, and centrifuge.	Remove 7.5 mL of the liquid to another tube with 750 mg primary–secondary amine (PSA): aminopropyl (NH2) (50:50, m/m). Shake and centrifuge for 5 min at 0 °C. Transfer 6 mL of the supernatant and evaporate to below 1.1 mL. Add 0.4 mL of DMSO: MeOH (25:75, *v/v*) to the tube concentrate to 1.5 mL with water and shake for 20 s.	50	2017	4.8–16 µg/kg	60–139%	Doxycycline, tetracycline, ofloxacin, enrofloxacin, sulfaclozine, sulfaquinoxaline, and amantadine were found in 22 chicken samples. Two positive samples were found in chicken muscle containing sulfaclozine and sulfaquinoxaline at 4524 and 286 μg/kg, respectively, which exceeded the EU Maximum Residue Limits (MRL) (100 μg/kg). Amantadine was found in five chicken samples (58–927 μg/kg). Other drugs were lower than their corresponding EU MRL. The very hydrophobic analytes were not included in this study.	[75]
Nile tilapia	AM (CAP, FF, TAP)	SLE	1 g + 10 mL of ethyl acetate: ammonium hydroxide (98:2). Vortex, centrifuge 7000 g, 4 °C, 1 min. Add 500 µL of 5% acetic acid to the supernatants. Shake and evaporate to 1–2 mL at 55 °C. Add 250 µL of 5% acetic acid to the concentrated extract, vortex 30 s. Add heptane (5 mL), vortex 1 min, centrifuge at 7000g at 4 °C for 1 min. Discard the heptane phase. Evaporate to dryness.		32	2018	0.60–75 µg/kg	79.8–92.0%	One (3.1%) sample was positive for thiamphenicol <LOQ (12.5 μg/kg). All samples were positive for florfenicol at levels below the LOQ (12.5 μg.kg^−1^). The presence of florfenicol in every sample analyzed, even below MRL, suggests this drug has been used widely in tilapia production, not only in fish farming.	[76]
Muscle, kidney, liver	QN, SA, TC, MA, LS, CP, PN, AM, AG, TU, BA, AT, CD, NMZ, TQ, Anti-inflammatories and others (174)	SLE, SPE, d-SPE	Aminoglycoside: 2 g + 20 mL of 10 mM NH^4^OAc, 0.4 mM EDTA, 0.5% NaCl, and 2% TCA. Vortex for 5 min and centrifuge for 3 min at 4150 rpm. Transfer 10.75 mL (1 g of equivalent sample) of extract, adjust the pH to 6.5 ± 0.1 with a few drops of 30% aqueous NaOH solution. Centrifuge again. MMM (Multiclass, Multiresidues): 2 g + 10 mL of 4/1 (*v/v*) ACN/water. Vortex for 5 min and centrifuge for 3 min at 4150 rpm.	Aminoglycoside: Conditioning each WCX (50 mg) (Weak Cation Exchange) tip on the DPX (Dispersive Pipette Extraction) device with a few pumps of 3 mL MeOH + 3 mL of water sequentially. Repeatedly pull the 10.75 mL extracts to the DPX tips in four portions of ± 2.7 mL each. Wash the sorbents at the tips with 3 mL of water and dry. Aspirate 1 mL of 10% aqueous formic acid solution into and out of the tips five times. Eluate DPX tips with 1 mL of 10 % FA in water. Combine 407 μL of the MMM extract (0.172 g/mL of the equivalent sample) and 70 μL of the aminoglycoside extract (1 g/mL) in a 1 mL autosampler vial. Add 273 μL of 146.5 mM 1-heptanesulfonate aqueous reagent IP solution (ion-pairing reagent).		2017	0.5, 1, and 2 times the regulatory levels of interest (10–1000 ng/g, depending on the drug)	70–120% in 79–84% of the analyzed analytes	Bad results for aminoglycosides in muscle because the muscle extracts were very fatty and blocked the ends of the DPX during sample preparation. In the case of bovine muscle, 100/131 medications (76%) previously met the criteria for quantitative validation, and in this study, 137–149 of 174 medications (79–84% depending on the matrix) met the criteria.	[77]
Chicken Tissues	MA	SPE	2.0 g + 200 μL of 0.1 mol/L EDTA solution. Shake 20 min, centrifuge at 5000 r/min for 5 min. Extract the supernatant twice with 10mL of ACN/MeOH (*v/v*, 95:5). Evaporate to 1 mL after adding 0.4 g of NaCl. Wash with 1mL of acetonitrile and 15mL of water and collect the eluent.	The pretreatment procedure of PAF-6 SPE cartridge (60 mg/3 cc): first, the cartridges were prepared by packing 60 mg of PAF-6 into the empty polypropylene SPE cartridges (3 mL). Pass 8 mL of the extract through the cartridges, preconditioned with 3 mL of methanol + 3 mL of water. Wash with 5 mL. Elute with 5mL of 5% methanol ammonia. Evaporate and redissolve to 1.0 mL with the mobile phase.	6	2018	1–50 µg/kg	82–101.4%	Tylosin was detected in two samples with contents of 38.752 μg/kg and 79.211 μg/kg, respectively. Azithromycin and tilmicosin were detected in one sample; the contents were 27.336 μg/·kg and 56.719 μg/kg, respectively.	[61]
Pangasius fillet	PN, QN, MA, SA, TC, AM, trimethoprim	SLE	2.0 g + 0.5 mL 0.1 M EDTA. Vortex shake for 2 min. Add 3.5 mL of ACN, vortex; shake for 5 min. Centrifuged for 5 min at 13,000× *g* at 18 °C.		40	2019	Low: 3 ng/g Low middle: 10 ng/g High-middle: 50 ng/g High: 100 ng/g	Low: 80.7–119.8 % Low-middle: 78.8–118.3 % High-middle: 76.9–114.2 %	Samples showed enrofloxacin residue levels, all of which were above the LOD of the method (1.00 ng/g) but below the LOQ (3.00 ng/g). The analysis of pangasius samples imported from Vietnam and acquired in the Brazilian retail market indicated the presence of low residue levels of enrofloxacin in five out of 40 samples analyzed. Considering the FDA, Brazilian and Vietnamese regulatory framework, and the fact that there is no safe level (MRL) set by Codex Alimentarius for enrofloxacin residues in fish that may pose an acceptable risk for consumers, those contaminated samples must be considered out of conformity.	[37]
Muscle, Eggs	QN	SLE, SPE	5.0 g + 20 mL EDTA Mcllvaine buffer (0.1 mol/L, pH 4.0). Vortex shake. Add 10 mL of MeOH, centrifuged at 8000 rpm for 10 min at 4 °C. Filter the supernatant on a qualitative quick filter paper. Dilute the filtrate with 100 mL with water.	Conditioning Oasis HLB cartridges with 3 mL of MeOH + 3 mL of water. Pass 20 mL of the extract. Wash with 1 mL of 5% (*v/v*) MeOH in water, elute with 6 mL of MeOH. Evaporate and redissolve in 1.0 mL of 0.2% formic solution.	170	2019	2.0, 5.0 µg/kg	Muscle: 70.4–98.4% Egg: 66.9–99.0 %	Enrofloxacin was detected in six chicken meat samples, and its level varied from 4.88 to 44.4 µg/kg; traces of ciprofloxacin (lower than 7 µg/kg) were also found. In addition, eight of 110 egg samples contained traces of enrofloxacin at levels ranging from 1.09 to 5.22 µg/kg. Traces of ciprofloxacin were also present in egg samples (<7 μg/kg).	[64]
Honey	AG	SLE, d-SPE	0.2 g of sample + 2mL H_2_O. Vortex shake and ultrasound. Add 2ml of ACN, vortex; shake for 2 min, and centrifuge 20 min at 15 °C.	Magnetic Fe_3_O_4_@SiN- galactitol nanoparticles for DSPE (1 mg): Pass the supernatant in the DSPE. Elute with 150 µL FA 190mM		2019	15–60 µg/kg	84–109%	Compared to other SPE methods previously reported for AGs analysis, the present one employed a minimum amount of sorbent (1 mg) and sample (0.2 g). The final optimized method was validated for the analysis of four aminoglycosides in honey with acceptable and reliable results.	[78]
Duck meat	TT, SA, PP, QN, MA, LS, trimethoprim	SLE, SPE	2 g + 6mL of 0.1 M Na_2_EDTA solution + 4mL of 2% TFA solution. Vortex shake for 5 min and centrifuged at 2600 g at 4 °C for 10 min. The obtained supernatant was transferred to a new tube containing 10 mL of n-hexane, vortexed for 5 min, and centrifuged at 2600 g for another 10 min.	Conditioning the Oasis HLB columns (6 cm^3^, 200 mg) with 3 mL ACN + 3 mL MeOH + 2 mL of distilled water. Pass 10 mL of the extract. Wash the columns with twice 5 mL of distilled water and dry for 10 min. Elute with 3mL MeOH + 3 mL ACN + 3 mL of 0.02% ammonia solution in MeOH. Evaporate to dryness and redissolve in 2 mL of MeOH.		2019	5, 10, 20 µg/kg	69.8–103.3%	Values of LOD ranging from 1.63 to 8.65 μg/kg and LOQs ranging from 4.93 to 26.21 μg/kg were achieved. LOQ values were much lower than the MRLs. The matrix effect varied between −47.2% to −13.5%, and an ion suppression was observed for all analytes in the duck meat matrix.	[50]
Milk	BZM, CP, IZ, MA, NSAIDs, PN, QN, SA, BA, steroids (66)	d-SPE, SPE	Procedure I: 1 g + 4 mL ACN (2% FA). Mix in vortex. Centrifuge at 6460 g for 5 min. Procedure II: 2 g + 10 mL ACN (5% FA). Mix in vortex. Centrifuge at 6460 g for 5 min.	Procedure I: Conditioning the HLB PRiME columns (3 cc, Waters) with 3 mL of ACN (2% FA). Pass 4 mL of the organic layer. Dilute 100 μL of the eluted extract from the cartridge with 100 μL of ACN + 800 μL of H_2_O so that the final composition is 80:20 (*v/v*) aqueous: organic. Procedure II: Solvent extraction followed by EMR-Lipid dSPE cleaning (EMR-Lipid dSPE and EMR-Lipid Polish tubes, Agilent Technologies): Condition the EMR-Lipid dSPE sorbent with 5 mL of ammonium acetate buffer solution (5 mM). Add 5 mL of the organic layer to the EMR-Lipid dSPE tube. Shake manually for 1 min and vortex for 1 min. Centrifuge at 2650 g for 3 min. Add 5 mL of the top layer of the tube to the EMRLipid Polish tube (containing 1.6 g of MgSO_4_ + 0.4 g NaCl). Vortex for 2 min. Centrifuge at 2650 g for 3 min. Collect the organic phase. Make a final 1:10 dilution with a 100 μL aliquot of the extract, resulting in a final composition of 80:20 (H2O/ACN, *v/v*).	24	2018	50 μg/kg	Procedure I- 72.4–115.9 % Procedure II: 75.6–119.2 %	Traces of danofloxacin were found in two whole cow milk, in the range of 0.7–1.5 μg/kg. However, these concentrations were below the current MRL established. Both methodologies provide satisfactory results in terms of matrix effect, sensitivity, recoveries, precision and being environmentally friendly. The HLB PRiME procedure is faster than EMR-Lipid. The tolerance to the matrix effect was higher with the SPE cleaning.	[79]
Meat (Pork)	AM, BA, BZM, CCD, CDC, IO, LS, MA, NMZ, QN, SA, TC, TQ, antivirus drugs, resorcylic acid lactones, steroid hormones, triphenylmethane dyes, and others	SLE, SPE	2.0 g + 0.5 mL 0.1 M EDTA + ACN/water solution (6 mL, 80/20, *v/v*). Shake end-over-end for 10 min and centrifuge at 4600 rpm for 10 min.	Conditioning C18 SPE cartridges (Waters, Milford, MA, USA) with ACN/H_2_O (3 mL, 80/20, *v/v*). Pass the extract and collect. Evaporate to dryness and re-dissolve in 500 μL of water-ACN solution mixture (95/5, *v/v*) containing 5 mM ammonium formate and 0.1% formic acid.	40	2020	LLOQ (µg/kg) = 10, 100 or LLOQ (µg/kg) = 50, 100	70% for all of the compounds with the exception of triamcinolone, triamcinolone acetonide, fluocinolone acetonide and clobetasol propionate.	Sulfamethazine was detected in one sample, and its metabolites were successfully found in one run. Sulfamethazine content (1150 µg/kg) was much higher than the MRL established in pork (100 µg/kg).	[80]
Muscle, Fat, Liver and Kidney	PN (Amoxicillin)	SPE	2.0 g + 8 mL phosphate buffer (0.01 M, pH = 6.3). Refrigerated centrifuge at 10,960 g for 10 min at 4 °C. Vortex for 2 min and refrigerated centrifuge at 10,960 g for 10 min at 4 °C after addition of 1 mL TCA solution (50 mg/mL).	Condition Oasis^®^ HLB cartridges (60 mg, 3 mL) with 3 mL of MeOH + 3 mL of water. Pass the extracts. Wash the columns with 2 mL of water. Elute with 3 mL of ACN. Evaporate to dryness and re-dissolve with 2 mL of initial mobile phase.			Muscle, fat, liver, kidney: 10–100 µg/kg	Muscle (µg/kg): 91.06–100.81 Fat (µg/kg): 102.39–101.79 Liver (µg/kg): 113.94–111.72 Kidney (µg/kg): 98.67–89.49	The results obtained from the present study revealed satisfactory recovery and precision, which were consistent with the EU requirements, indicating that the method using stable isotopically labeled analogs as an internal standard was reliable.	[81]

**Table 3:** The main extraction techniques for determination of antibiotic residues in foodstuffs under the concept of green chemistry methods (greenness factors): advantageous factors are shown in green, factors with limitations in yellow and disadvantageous factors are shown in red.

	SPME	SBSE	MSPD	Micro-SPE	SPE	d-SPE	d-SPME	MSPE	MIP-SPE	LLE	QuEChERS
Sensitivity	high	higher (SPME)								high	
Automation	convenient		difficult								
Extraction Time/Sample preparation	short	long	short	short	short	extremely fast				fast	fast
Efficiency of Extraction		High control			may be affected by poor packing		high efficiency	high efficiency			high efficiency
Cost	low				low		low	low	low		low
Coupling with chromatographic instruments	easy				easy		highly compatible (HPLC)				
Solvent consumption	low		low	low	possibility		minimal	minimal		large	
Simplicity	reduced labor-intensive manual operations		yes		yes		yes	easy to perform	easy to perform		easy to perform
Single step	sampling and extraction		extraction and cleaning-up	extraction and concentration							extraction and cleaning-up
Sorbent choice	difficult	limited availability			broad variety			easy to perform			
Robustness/Reproducibility	low					yes, to different types of samples and analytes			low		high
Stationary phase, when exposed to organic solvents	instability										
Loading of analyte into the sorbent	limited	higher							limited to polar compounds		
Thermal stability of physically holding sorbent	low										
Diffusion of the analytes into viscous sorbents	slow	slow									
Lifetime of the physically holding sorbent	short										
Matrix Effects	Selective for target analytes	strong			high	decrease analyte recovery					strong
Recovery (of target analyte)				high	sometimes low	high			sometimes low	high	
Nº of available stationary phases/Fragility of fiber/Analyte carryover/Sorbent dispersion				limited/yes/possible			impaired by addition of salt; lower at high pH values				
Additional clean-up steps					needed (to reduce the number of interferers	needed (to reduce the number of interferers					
Capacity and dispersibility of sorbent in liquid samples							High				

**Table 4:** Analytical methods for determination of antibiotic residues in foodstuffs. References are in chronological order for evaluation of the analytical trends.

Antibiotics Residues Analyzed	Detector	Conditions	Analytical Column	Internal Standard	Ccalfa and Ccbeta	References
PP	UHPLC-MS/MS (Acquity UPLC) coupled to a TSQ Quantum Access Max triple-quadrupole MS, (Thermo Fisher, San Jose, CA, USA)	Mobile phase: A- 50 mL ACN + 3 mL of FA + 0.1 mL of TCA into a 1000 mL volumetric flask and diluted to volume with purified water. B- 50 mL of purified water + 3 mL of FA + 0.1 mL of TCA into a 1000-mL volumetric flask and diluted to volume with ACN. Gradient program: 0–2 min with 8% B and flow 0.4 mL/min, 2–7 min with 8–20% B, 7–8 min with 20–30% B, 8–11 min with 30–100% B, 11–11.1 min with 100% B and flow 0.4–0.8 mL/min, 11.1–12.5 min with 100%, 12.5–12.51 min with 100–8% B and flow 0.8–0.4 mL/min, 12.51–14 min with 8% B. Flow-rate: 0.4 mL/min Ionization: ESI source in the positive mode Temperature column: 30 °C Injection volume: 20 μL Capillary voltage: 3 kV Collision gas (argon) pressure: 1.5 mtorr Vaporizer temperature: 380 °C Sheath gas pressure: 45 units Auxiliary gas flow: 10 units	Kinetex C-18 column (2.1 × 150 mm × 2.6 µm) with an installed pre-filter (Krudkatcher), both from Phenomenex (Torrance CA, USA)		Muscle LOD (µg/kg) = 5–30 CCα (µg/kg) = 8.6–177.5 CCβ (µg/kg) = 14.7–251.33 Kidney LOD (µg/kg) = 15–30 CCα (µg/kg) = 9.5–241.8 CCβ (µg/kg) = 16.2–358.5	[87]
BZM, AT	UHPLC-MS/MS (Acquity UPLC) coupled to a TSQ Quantum Ultra AM triple-quadrupole MS (Thermo Fisher, San Jose, CA, USA) equipped with electrospray (ESI), atmospheric pressure chemical ionization (APCI) and atmospheric pressure photoionization (APPI)	Mobile phase: A- 0.1% FA; B-ACN Gradient program: first, an isocratic step at 5% solvent B was held for 0.7 min and then the organic modifier percentage increased to 25% during 1.3 min, in a third stage solvent B was raised to 40% in 2 min and to 100% in 4 min more and held for 1 min at this percentage before returning to the initial settings Column temperature: 25 °C Flow rate: 500 µL/min Injection volume: 10 μL Ion-transfer tube temperature: 200 °C APCI Current discharge: 15 µA Vaporizer temperature: 300 °C Voltage: 4 kV for ESI; 10 eV (krypton lamp) for APPI Dwell time: 50 ms Collision gas: 1.5 mTorr Collision energy (CE): 5–35 eV Data acquisition: selected reaction monitoring (SRM)	Ascentis Express C18 column (150 mm × 2.1 mm, 2.7 µm, superficially porous particles) from Supelco	Mebendazole-d3	LOD (µg/Kg) = 1–10 LOQ (µg/Kg) = 0.6–1.5	[70]
AM, AV, BZM, BA, PN, CP, DYE, IO, LS, MA, NMZ, NSAIDs, PP, AM, QN, SA, TC, Miscellaneous, Flukacides (150)	LC-MS/MS coupled to a triple quadrupole time-of-flight (Q-TOF) 6530 MS (Agilent, Santa Clara, CA, USA)	Mobile phase: 0.1% FA (A) and ACN (B) Gradient program: 5% ACN for 2 min, ramp 5−50% ACN over 10 min, hold at 50% ACN for 1 min, ramp 50−100% ACN over 3 min, hold at 100% ACN for 2 min. For most analytes, the column was held at 100% ACN for the 2 min described above for a total analysis time of 18 min. However, some compounds being investigated did not elute in that time frame, so an additional 4 min of 100% ACN can be added to the chromatographic program to make an extended LC program that is 23 min long Flow rate: 0.25 mL/min Ionization: ESI Agilent Jet Stream Technology Column temperature: 22 °C Injection volume: 10 μL Fragmentor: 150 V, nozzle: 250 V, Vcap: 4000 V, drying gas (N2): 11 L/min, sheath gas (N2): 11 L/min TOF (MS1 only) data acquisition rate: 4 GHz (to m/z 1700); Scan range: m/z 100−1200 at 1.08 spectra/s Compounds that ionized in the negative ion mode were analyzed separately using a different acquisition program. The MS source parameters were as above for negative ions except that the Vcap and nozzle voltages were 2000 and 200 V, respectively. MS/MS data were collected at three different collision energies (CEs) for each compound. The formula “[3 × (mass/100)] + 10” is suggested by Agilent to calculate a reasonable CE for compounds using their acquisition software and was used here; data were also collected at approximately 10 V higher and lower than that value. Medium isolation width (4 m/z) and 200 ms/spectrum were used to collect product ion spectra.	YMC ODS-AQ (120 Å, 2 100 mm, 3 μm) from Waters Corp. (Milford, MA, USA).	Chloramphenicol-d5	Estimated screening, LOD—10–100 ng/L	[107]
MA, LS	LC–MS/MS, LC (Agilent 1100 Series) and an API 5000 MS (AB Sciex, Foster City, CA).	Mobile phase: A- 0.1% FA; B- ACN with 0.1% FA Gradiente program: starts keeping 98% of A during 1 min, and then decreasing linearly to 5% of A during 4 min. This condition is held for 3 min. Finally, A% increases linearly until 10 min, returning to 98%, and this condition is kept for 2 min, with a total run time of 12 min. Between each analysis, 3 min of equilibration time is applied, using the initial gradient conditions (98% A) Flow rate: 0.3 mL/min Source temperature: 550 °C Curtain gas (CUR): 20 ps Ion spray voltage (IS): 5500 V Ion source gas 1 (GS1): 55 psi, Gas 2 (GS2): 45 psi Interface Heater on Collision gas (CAD): 4 mTorr Entrance potential (EP): 10 V Dwell time: 100 ms Data acquisition: MRM mode	HPLC column AgellaDurashell RP (100 mm × 2.1 mm, 5 μm); guard column filled with C18 (4.0 mm × 3.0 mm, 5 μm, from Phenomenex)		Milk LOD (µg/L) = 5–25 LOQ (µg/L) = 10.0–50.0 CCα (µg/L) = 50.2–230.0 CCβ (µg/L) = 60.5–272.1 Muscle (Bovine) LOD (µg/kg) = 6.2–12.5 LOQ (µg/kg) = 12.5–25.0 CCα (µg/kg) = 57.9–120.9 CCβ (µg/kg) = 65.7–149.5	[31]
Amoxicillin and metabolites	LC-MS/MS, Agilent 1200 LC system (Agilent Technologies Inc., Palo Alto, CA, USA) coupled with a 6460 triple quadrupole MS (Agilent, Palo Alto, California, USA)	Mobile phase: A- ACN; B- 0.1% FA in water Gradient program: time 0, 100% B; 2 min, 98% B; 5 min, 80% B; 12 min, 50% B; 20 min, 98% B Flow rate: 1 mL/min Injection volume: 20 μL Ionization: ESI source in positive mode Capillary voltage: 4 kV Gas temperature: 300 °C Gas flow: 10 L/min Nebulizer gas: 15 ps Sheath gas temperature: 250 °C Sheath gas flow: 7 L/min Data acquisition: MRM mode	Agilent C18 column (50 mm × 4.6 mm; 5 µm) protected with a guard column (C18; 5 µm)	Penicillin V	LOD (µg/kg) = 0.1–0.6 LOQ (µg/kg) = 0.3–0.8 µg/kg CCα (µg/kg) = 11.1–11.15 µg/kg CCβ = 12.1–13.0 µg/kg	[58]
PN, SA, TC, MA, LS, QN, AM, PT, DP, Rifamycines (62)	High resolution LC-MS. Thermo Ultimate 3000 High Performance LC system (San Jose, CA, USA). Mass spectrometer Q-Orbitrap (Thermo Scientific, San Jose, CA, USA) was coupled with heated ESI (HESIII) source.	Mobile phase: A- aqueous solution containing 0.1% (*v/v*) FA; B- MeOH Gradient program: initiated with 5% eluent B for 1 min, continued with linear increase to 95% B in 19 min. This condition was maintained for 5 min. The system returned to 5% B in 1 min and was re-equilibrated for 4 min (run time: 30 min) Flow rate: 0.25 mL/min Column temperature: 30 °C HESI-II temperature: 320 °C Capillary temperature: 300 °C Electrospray voltage: 3.00 kV (positive mode) Sheath and auxiliary gas: 35 and 15 arbitrary units. Acquisition: full scan/dd-MS2; Mass range in full scan was within m/z 150–1200. The data were acquired at a resolution of 70,000 FWHM (m/z 200).	Poroshell 120 EC-C18 column (100 × 3.0 mm; 2.7 µm); guard column Poroshell (2.1 × 5 mm)	Penicillin G-d7 spiramycin I-d3 tetracycline-d6 florfenicol-d3 sulfamethazine-^13^C6 sulfanilamide-^13^C6 enrofloxacin-d5 cefadroxil-d4	Muscle (bovine) CCα (µg/kg) = 1–1128.0 CCβ (µg/kg) = 3.3 -1272 Muscle (swine) CCα (µg/kg) = 1–1374 CCβ (µg/kg) = 3.3–1573 Muscle (poultry) CCα (µg/kg) = 1–441 CCβ (µg/kg) = 3.3–486	[108]
AG, PN, MA, SA, AT, CD, NSAIDs, Pharmaceuticals, Rifaximin, Baquiloprim, Trimethoprim, Chlorpromazine, 6-phenyl-2-thiouracil, Bromhexine (76)	UHPLC-MS/MS. Thermo UHPLC Accela system (Thermo, San Jose, CA, USA) coupled to a Thermo Scientific TSQ Quantum Access Triple Quadrupole MS, USA)	Mobile phase: A- ACN, B- aqueous ammonium formate 1 mM with 0.1 FA (*v/v*), C- MeOH Gradient program: 80% A and 20% B (0%C), decreased linearly to 0% A and 95% B (5%C) in 10 min and was held for additional 4 min. Total run time: 20 min Injection volume: 10 µL Spray voltage: 4000 V Sheath gas: 25 psi Auxiliary gas: 10 a.u. Capillary temperature: 300 °C. Data acquisition: reaction monitoring (SRM) mode	ACQUITY UPLC BEH HILIC (100 mm × 2.1 mm, 1.7 µm, Waters)	Nigericin amikacin flubendazole flunixin d3 meloxicam d3 sulfadiazine d4 sulfadimidine d4 sulfadimethoxine d4	LOQ (µg/kg) = 0.03–178 CCα (µg/kg) = 2.2–1151 CCβ (µg/kg) = 2.4–1302	[72]
QN, MA, PN, NMZ, SA, LS, AM, QNX, TC, PP, tiamuline, bacitracin, flavomycin, antibacterial synergists, including Diaveridine (DVD), Trimethoprim (TMP). (12 classes of drugs, 120 compounds)	HPLC-MS/MS. Finnigan HPLC system (Thermo Fisher Scientific, Waltham, MA, USA) coupled to TSQ Quantum Access triple quadrupole MS (Thermo Fisher Scientific).	Mobile phase: A- 0.1% FA in water, B- ACN Gradient program: 0–5 min, 0.1 mL/min, 5% B; 5–10 min, 0.2 mL/min, linear increase to 25% B, and hold for 15 min; 25–40 min, 0.2 mL/min, increase to 50% B; 40–45 min, increase to 80% B, hold for 5 min; 50–50.5 min, decrease to 5% B, hold for 9.5 min. Auxiliary gas: N2 at a pressure of 5 arbitrary units Collision gas: Argon used for collision induced dissociation at a pressure of 25 arbitrary units. Column temperature: 40 °C Injection volume: 10 µL Spray voltage: 4.5 KV Capillary temperature: 350 °C	Hypersil Gold C18 (150 × 2.1 mm, 5 µm) column		LOD (µg/kg) = 0.5–3.0 LOQ (µg/kg) = 1.5–10.0	[96])
SA, QN	UHPLC-MS/MS. Waters ACQUITY UPLC system was coupled to a Xevo triple quadrupole MS (Waters, Milford, MA, USA).	Mobile phase: A-MEOH; B- 0.1% FA in water Gradient program: 0.0% A; 0–2 min, linear increase 25% A; 2–7 min, linear increase 30% A; 7.01–8 min, 45% A linear increase 60% A, and hold for 3 min; 11.01–12 min, 100% A hold for 1 min; 12–14 min, decrease 10% A, and hold for 2 min. Flow rate: 0.2 mL/min Injection volume: 10 μL Ionization: ESI in the positive mode Capillary voltage: 3 kV Source temperature and desolvation temperature: 150 and 400 °C, respectively. Data acquisition: MRM mode	Waters Acquity UPLC CORTECS C18 column (150 mm × 2.1 mm, 1.6 μm)		LOD (µg/kg) = 0.05–2.6 LOQ (µg/kg) = 0.12–5.6	[68]
TC (5) and epimers TC’s (5)	LC-MS/MS. Agilent 1290 Infinity LC system (Waldbronn, Germany) coupled to a QTrap 5500 triple quadrupole fitted with the Turbo V™ ion source (Sciex, Toronto, Canada).	Mobile phase: A- 0.01 M oxalic acid, B- MEOH containing 0.1% FA Gradient program: 11-min gradient was set as follows: 0–0.5 min (1% B); 0.5–2 min (35% B); 2–3.5 min (55% B); 3.5–6 min (55% B); 6–6.5 min (100% B); 6.5–8 min (100% B); 8–8.5 min (5% B); 8.5–11 min (5% B). Flow rate: 0.4 mL/min Injection volume: 10 μL Ionization: ESI source in positive mode Temperature (TEM): 550 °C Curtain gas (CUR): 30 psi Nebulising gas (GS1): 40 psi Drying gas (GS2): 40 psi Collision activated dissociation (CAD) gas pressure: Medium. Ion spray voltage: 5000 V Data acquisition: MRM mode	Acquity UPLC HSS T3 column (2.1 × 100 mm, 1.8 µm), guard column: HSS T3 VanGuard pre-column (2.1 × 5 mm, 1.8 µm)		STC (µg/kg) = 50	[45]
PN, CP, SA, TC, MA, LS, QN, AM, PT, PP, DP, Novobiocin, Rifaximin, Virginiamycin M_1_ (75)	LC-MS/MS Shimadzu LC20AD-XR system (Kyoto, Japan) coupled to API4000 MS (ABSciex, San Jose, CA, USA)	Mobile phase: A-mixture of 1 mmol/L HFBA and 9.5 mmol/L PFPA in water; B- mixture of 1 mmol/L HFBA and 9.5 mmol/L PFPA in ACN Gradient program: started with 10% B. It was then raised to 30% B over 4 min, then held for 1 min and again raised linearly to 70% B over 2 min and held for 3 min. The initial composition was then recovered over a 1-min delay. Flow rate: 0.60 mL/min Ionization: ESI source in positive mode Source temperature: 700 °C Turbo-ion-spray voltage: 5500 V Sheath gas pressure (air): 40 psi Auxiliary gas pressure (air): 50 psi Curtain gas: 20 psi Data acquisition: MRM mode	Waters Symmetry C18 column (150 mm × 3.9 mm, 5 µm), protected with a C18 security guard system from Phenomenex (4.0mm × 3.0mm, 5 µm)	Sulfaphenazole	Cval (µg/kg) = 50–400	[52]
TT, NMZ, LS, AT, TC, PN, QN, BA, CP, SA, MA, AM, CDC, TQ, Flukicide, NSAID, Progestin	UHPLC-MS/MS. UHPLC system was coupled to a triple quadrupole MS model 6490 system (Agilent Technologies, Santa Clara, CA, USA) equipped with electrospray Jet Stream, and iFunnel technology.	Mobile phase: A- 0.1% FA in water; B- 0.1% FA in ACN Gradient program: 10% B for 0–0.5 min, then to 100% B by 8 min, and hold 100% B until 12 min, followed with a post column equilibrium time for 3 min Flow rate: 0.3 mL/min Column temperature: 30 °C Injection volume: 3 µL Gas temp: 120° C Gas flow: 14 L/min Capillary voltage: 3000 V Nebulizer pressure: 40 psi Sheath gas heater: 400 °C Sheath gas flow: 12 L/min Nozzle voltage: 0 V for both positive and negative ion mode iFunnel parameters were: high pressure RF of 90 V for positive and negative ion mode; and low pressure RF of 70 V for positive and 60 V for negative ion mode Data acquisition: MRM	Poroshell 120 EC-C18 column (150 × 2.1 mm, 2.7 µm), Poroshell 120 guard column (5 × 2.1 mm, 2.7 µm) (Agilent, Newport, DE, USA)	Flunixin-d3 (NEG) Flunixin-d3 (POS)	LOQ (µg/kg) = 1–5	[51]
AM, AV, BZM, CCD, LS, MA, NSAIDs, QN, SA, TQ, Other antibiotics (105)	LC-MS/MS. Agilent 1290 Infinity LC system (Waldbronn, Germany) coupled to a QTrap 5500 triple quadrupole (Sciex, Toronto, Canada)	Mobile phase: A- water, B- MEOH) both containing 0.5 mM of ammonium formate and 0.1% FA Gradient program: 1- Multifamily run (16 min): 0–0.5 min: 5% B; 0.5–2.5 min: linear gradient to 35% B; 2.5–10 min: linear gradient to 100% B; hold at 100% B for 2 min; return to 5% B in 0.5 min and hold at 5% B for 3.5 min Flow rate: 0.4 mL/min Injection volume: 10 µL LC flow directed into the MS detector between 0.5 and 12 min using the integrated diverter valve Ion source: 550 °C Curtain gas (CUR): 30 psi Nebulising gas (GS1): 40 psi Drying gas (GS2): 40 psi Collision activated dissociation (CAD) gas pressure set as medium Ion spray voltages (IS): 5000/−4500 V. Gradient program: 2- Avermectins run (10 min): 0–0.2 min: 5% B; 0.2–1 min: linear gradient to 75% B; 1–4.5 min: linear gradient to 100% B; hold at 100% B for 2 min; return to 5% B in 0.5 min and hold at 5% B for 3 min. Elution conditions: 0.4 mL/min Injection volume: 10 µL Avermectins Ion source: 350 °C The other parameters were kept. Data acquisition: MRM mode	Acquity BEH VanGuard pre-column (2.1 × 5 mm, 1.7 µm) and Acquity BEH C18 LC column (2.1 × 100 mm, 1.7 µm) (both from Waters, BadenDättwil, Switzerland)		STC (µg/Kg) = 0.3–10	[98]
AV, BZM, BA, CP, CAP, FF, DYE, HOM, IZ, MA, QN, QX, TQ, SA, TC, organochlorines, organophosphates, others (>200)	High resolution LC-MS. Ultrahigh-performance liquid chromatography (UHPLC) system (DionexUltiMate 3000, Thermo Fisher Scientific) coupled to a quadrupole-Orbitrap MS with ESI (Q-Exactive, Thermo Fisher Scientific)	Mobile Phase:A- 0.1% FA in ACN, B- 0.1% FA in water Gradient program: 5% (A) 0.1% FA in ACN for 3 min, then linearly increased to 100% in 19min, and kept constant for 3 min. In the end, the eluent was restored to the initial conditions for 5 min to re-equilibrate Flow rate: 0.3 mL/min Injection volume: 10 µL Ionization: ESI in positive and negative mode Spray voltage: 3200 V (positive mode), 2800 V (negative mode) Sheath gas flow rate: 8 L/min Auxiliary gas flow rate: 10 L/min Sweep gas flow rate: 1.5 L/min Capillary temperature: 325 °C S-lens RF level: 60 V Data acquisition: Full MS/ddMS2 (with inclusion list/full-scan data dependent MS/MS) mode over the mass range m/z 100–1000 (positive mode) and 150–1000 (negative mode)	Accucore RP-MS C18 column (2.1 × 100 mm, 2.6 μm, Thermo Fisher Scientific, USA)		SL (carp, shrimp, crab, eel, mussel) (µg/kg) = 1–50	[106]
AM (CAP, FF, TAP)	LC-MS/MS. Agilent 1200 Series HPLC (Agilent Technologies Inc., Santa Clara, CA, USA) coupled to a Triple Quadrupole MS detector QTRAP API 5500 Sciex (Framingham, MA, USA)	Mobile phase: A- 0.1% of FA in water, B- 0.1% of FA acid in MEOH Gradient program: 90% A; 0.5 min–90% A; 2.0 min–70% A; 7.0 min–90% A; and 9.0 min–90% A; Total run time: 9 min. Flow rate: 400 µL/min Injection volume: 10 µL Column temperature: 40 °C	Aqua C18 column (50 × 2.0 mm, 5.0 μm, Phenomenex, Inc., Torrance, USA)	Deuterated chloramphenicol (CAP-d5)	LOQ (µg/kg) = 0.15–12.5 CCβ (μg/kg) = 0.068–58.91	[76]
AG, PP	High- resolution LC-MS. Thermo Ultimate 3000 High Performance LC system (Thermo Scientific, San Jose, CA, USA) coupled with mass spectrometer Q-Orbitrap (Q-Exactive, Thermo Scientific, San Jose, CA, USA) equipped with heated ESI (HESI-II) source.	Mobile phase: A- aqueous solution containing 1% (*v/v*) FA (FA) and 1mM ammonium formate (AF), B- ACN Gradient program: 20% eluent A for 2 min, continued with linear increase to 35% A in 5 min. In 1 min eluent A increased to 95%, and this condition was maintained for 7 min. The system returned to 20% B in 0.1 min and was re-equilibrated for 4 min (run time: 17 min). Column temperature: 40 °C Flow rate: 0.25 mL/min Injection volume: 5 µL HESI-II temperature: 300 °C Capillary temperature: 250 °C Electrospray voltage: 3.00 kV (positive mode); S-lens value: 50 V Sheath and auxiliary gas: 35 and 25 arbitrary units, respectively.	InfinityLab Poroshell 120 HILIC column (100 × 2.1 mm, 2.7 µm, Agilent Technologies, Santa Clara, CA, USA) connected with the InfinityLab Poroshell 120 HILIC guard column (5 × 2.1 mm, 2.7 µm)	Ribostamycin	Muscle CCα (µg/kg) = 10–1164 CCβ (µg/kg) = 10–1355 Milk CCα (µg/kg) = 10–167 CCβ (µg/kg) = 10–1860	[88]
SA, PN, CP, TT, PT, AM, LC, MA, DP, QN (60)	LC-MS/MS. Surveyor LC pump, coupled with a triple quadrupole mass spectrometer (TSQ Quantum Ultra, Thermo Fisher, San Jose, CA, USA)	Mobile phase: A- aqueous solution 0.1% (*v/v*) FA; B- MEOH Flow-rate: 0.25 mL/min Gradient program: 2% B for 1 min and increased linearly up to 95% B in 19.5 min; this condition was maintained for 5 min before returning to initial condition in 1 min (2% B) and held for 4 min to equilibrate the column. Ionization: ESI source in positive mode Column temperature: 30° C Injection volume: 10 µL Capillary temperature: 300 °C Vaporizer: 320 °C Spray voltage: 3 kV Sheath gas pressure: 35 units Auxiliary gas (nitrogen) pressure: 35 units Collision gas (argon) pressure: 1.5 mtorr Data acquisition: selected reactions transitions (SRM) monitored	Poroshell 120 EC-C18 column (3.0 × 100 mm; 2.7 µm). Guard column Poroshell (2.1 × 5 mm), both from Agilent Technologies (Santa Clara, CA, USA)	sulfanilamide-13C6 sulfamethazine-13C6 enrofloxacin-d5 florfenicol-d3 spiramycin-d3 metacycline	Muscle (bovine, porcine, ovi-caprine, rabbit, equidae) CCα (µg/kg) = 10 -1159 CCβ (µg/kg) = 10 -1349 Muscle (poultry) CCα (µg/kg) = 10 -448 CCβ (µg/kg) = 10 -502 Muscle (aquaculture) CCα (µg/kg) = 10 -1262 CCβ (µg/kg) = 10 -1594 Milk CCα (µg/kg) = 10 -239 CCβ (µg/kg) = 10 -286	[86]
AM, AT, AV, BA, CP, CCD, IO, LS, NMZ, NSAIDs, PN, QN, SA, TC, TQ, QNX, CDC, Insecticides, Estrogens, Androgens, Other antibiotics (164)	High resolution UHPLC-MS. Dionex UltiMate 3000 UHPLC system (Thermo Fisher Scientific, San Jose, CA, USA) coupled with Q-Orbitrap HRMS (Thermo Fisher Scientific)	Mobile phase: (A) 0.1% FA in water, (B) 0.1% FA in ACN, and (C) 0.1% FA in MEOH Gradient program: Flow rate: 0.2 mL/min: 90% of solvent A and 10% C (0% B) 0 min., 90% of solvent A and 10% C (0% B) (0.5 min.); 30% of solvent A and 70% C (0% B) (4 min); Flow rate: 0.3 mL/min: 20% of solvent A, 10% B and 70% C (5 min); 20% of solvent A, 70% B and 10% C (12 min); 0% of solvent A, 70% B and 30% C (15 min); 0% of solvent A, 70% B and 30% C (18 min.); Flow rate: 0.2 mL/min: 90% of solvent A, 0% B and 10% C (20 min); 90% of solvent A, 0% B and 10% C (25 min). Injection volume: 10 µL Column temperature: 40 °C Ionization: Electrospray voltage 4.0 kV in positive and 3.5 kV in negative ionization modes Heater temperature 350 °C Capillary temperature: 300 °C Sheath gas (N2): 20 arbitrary units (arb) Auxiliary gas (N2): 6 arb, S-lens RF: 50 arb. Data acquisition: Full scan data both in the positive and negative ionisation modes Resolving power: 70,000 FWHM	Phenomenex Luna Omega C18 analytical column (100 × 2.1 mm, 1.6 µm)	Albendazole-d3 Azaperone-d4 Chloramphenicol-d5 Cyromazine-d4 Diclofenac-13C6 DNC-d8 Enrofloxacin-d5 Flunixin-d3 Megestrole acetate-d3 Nigericin Toltrazuril-d3 Triclabendazole-d3 β-Testosterone-d2	CCβ (µg/kg) = 0.038–547.0	[101]
AM (CAP, FF, TAP)	LC-TOF-MS. Agilent 1290 Infinity II Series HPLC (Agilent Technologies, Santa Clara, CA, USA) coupled to an Agilent 6550 QTOF mass spectrometer, using an Agilent jet stream dual ESI (AJS-Dual ESI) interface.	Mobile phase: 70:30 water:MeOH (*v/v*) mixture under isocratic conditions Flow rate: 0.4 mL/min Ionization: ESI in negative mode Nebulizer gas pressure: 30 psi Drying gas: 130 °C Sheath gas: 300 °C Capillary spray, nozzle, fragmentor and octopole 1 RF Vpp voltages: 4000, 500, 360 and 750 V, respectively Collision energy: 40 V carried out with 2 GHz extended dynamic range mode and 3 spectra/s, 333.3 ms/spectrum and 1999 transients/spectrum. Reference mass of 112.985587 and 922.0098	Zorbax RRHD Eclipse Plus C18 (100 × 2.1 mm, 1.8 μm, Agilent)		LOD (pg/L) = 29.6 (TAP) LOD (pg/L) = 3 (FF) LOD (pg/L) = 3 (CAP)	[109]
PN, QN, MA, SA, TC, AM, DP	UHPLC-MS/MS. UHPLC system was coupled with an Agilent 6460 triple quadrupole tandem MS	Mobile phase: A- 0.1% FA solution, B- ACN acidified with 0.1% FA. Gradient mode: starting with 20% B for 2 min, increasing linearly until 90% over 8 min, remaining constant for 2 min and then returning to the initial proportion. A post-run interval of 5 min was necessary to re-equilibrate the column to the initial conditions. Flow rate: 0.5 mL/min Column temperature: 30 °C Injection volume: 20 μL Ionization: ESI source in the negative mode for the amphenicols and in the positive mode for the remaining compounds Source Temperature: 300 °C Gas flow:12 L/min Nebulizer gas: 30 psi Sheath gas flow: 12 L/min Sheath gas temperature: 350 °C Capillary voltage: 4.0 kV (positive mode) and 3.5 kV (negative mode) Nozzle: 1.5 kV Data acquisition: MRM mode	Agilent Zorbax SB C18 (4.6mm × 150 mm, 3.5 μm) column (Agilent Technologies, CA, USA)	1-penicillin G-d7 2-enrofloxacin-d5 3-roxythromycin 4-sulfadoxine-d3 5-chloramphenicol-d5 6-Oxytetracycline 7-Sulfathiazole 8-Enrofloxacin-d5, at concentration 50 ng/g	LOD (µg/kg) = 0.1–0.3 LOQ (µg/kg) = 0.3–10 CCα (µg/kg) = 0.37–1008.34 CCβ (µg/kg) = 0.44–1016.68	[37]
PN (amoxicillin)	LC-MS/MS. Shimadzu LC system (LC-30CE; Japan) consisted of a triple quadrupole MS (AB SCIEX Triple Quad™ 5500, AB SCIEX Corp., Framingham, MA, USA)	Mobile phase: A- 0.1% FA in water; B- 0.1% FA in ACN Gradient mode: 0–1.5 min, 95% A; 1.5−3 min, 70% A; 3–3.5 min, 10% A; 3.5−6 min, 95% A Gradient elution: 0.3 mL/min Column temperature: 30 °C Injection volume: 5 μL Ionisation: ESI in positive mode Spray voltage: 5500 V Temperature of ion source: 600 °C Pressures of spray gas: 60 psi Auxiliary gas: 60 psi Curtain gas: 35 psi Data acquisition: MRM mode	Luna Omega C18 column (50 mm × 2.1 mm, 1.6 μm)	Amoxicillin-D4	Muscle, Liver, Fat, Kidney LOD (µg/kg) = 5 LOQ (µg/kg) = 10	[81]
SA, TQ, NMZ, QN, PT, PN, BZM (78)	UHPLC-MS/MS. Waters Acquity ultra performance liquid chromatography system coupled to Micromass Xevo TQ-S triple quadrupole MS (Waters, Manchester, UK)	Mobile phase: A- 0.1% FA, B- MeOH:ACN, 2:8, *v/v*, containing 0.1% FA Gradient program: 0–1.0 min, 97% A; 1.0–4.5 min, 97–83% A; 4.5–6.0 min, 83–55% A; 6.0–6.5 min, 55–0% A; 6.5–7.5 min, 0% A; 7.5–7.6 min, 0–97% A; 7.6–9.5 min, 97% A Elution conditions: 0.3 mL/min Capillary voltage: 2.5 kV Source temperature: 150 °C Desolvation temperature: 500 °C Desolvation gas flow rate: 800 L/h Cone gas flow rate: 150 L/h Collision gas: argon at 0.15 mL/min Data acquisition: MRM mode	Acquity UPLC BEH C18 column (100 × 2.1 mm, 1.7 μm; Waters, Milford, MA, USA)		LOQ (µg/kg) = 0.1–1	[105]

## Data Availability

Not applicable.

## Abbreviations

ACN	Acetonitrile
AG	Aminoglycosides
AM	Amphenicols
AMX	Amoxicillin
AR	Antibiotics residues
AT	Anthelmintics
AV	Avermectins
BA	β-Agonists
BZM	Benzimidazole
CAP	Chloramphenicol
CC alfa	Decision limit
CC beta	Detection capability
CCD	Coccidiostats
CDC	Glucocorticoids
CL	Chemiluminescence
CP	cephalosporines
CTC	Chlortetracycline
Cval	Validation levels
DC	Doxycycline
dDMIP	Dummy molecularly imprinted polymer
DP	Diaminopyrimidines
DPX	weak cation exchange cartridges
dSPE	Solid-phase dispersion
d-SPME	Dispersive solid-phase microextraction
DYE	Dyes
EAEU	Eurasian Economic Union
EEA	European Economic Area
EMR-L	Enhanced Matrix Removal for Lipid
EFSA	European Food Safety Authority
ELISA	Enzyme-Linked Immune Sorbent Assay
EDTA	Ethylenediaminetetraacetic acid
EU	European Union
FA	Formic Acid
FDA	Food and Drug Administration
FF	Florfenicol
FVO	Food and Veterinary Office
GCC	Gulf Cooperation Council
HCL	Hydrochloric acid
HFBA	Heptafluorobutyric acid
HILIC–MS/MS	Hydrophilic Interaction Liquid Chromatography tandem Mass Spectrometry
HLB	Hydrophilic-lipophilic balance
HLB PRiME	Hydrophilic-lipophilic balance PRiME (process, robustness, improvements, matrix effects, ease of use)
HOM	Hormones
HPLC	High Performance Liquid Chromatography
HRMS	High-Resolution MS detectors
IO	Ionophores
IP	Ion Pairing
IZ	Imidazoles
LC	Liquid Chromatography
LC-MS/MS	Liquid Chromatography tandem Mass Spectrometry
LLE	Liquid-liquid extraction
LOD	Limit of detection
LOQ	Limit of quantification
LS	Lincosamides
MA	Macrolides
MAE	Microwave-assisted extraction
MEOH	Methanol
MEPS	Microextraction by packed sorbent
Micro-SPE MSPD	Micro-solid-phase extraction Matrix solid-phase dispersion
MIP	Molecularly imprinted polymer
MIP-SPE	Molecularly imprinted polymer solid-phase extraction
MISPE	Molecularly imprinted solid-phase extraction
MNPs	magnetic nanoparticles
MRL	Maximum residue limits
MS/MS	Tandem Mass Spectrometry
MSPE	Magnetic solid phase extraction
NMZ	Nitroimidazoles
OTC	Oxytetracycline
PDMS	Polydimethylsiloxane;
PEG	Poly ethylene glycol
PFPA	Pentafluoropropionic acid
PHWE	Pressurized hot water extraction
PLE	Pressurized liquid extraction
PN	Penicillins
PP	Polypeptides (Polymyxins)
PSA	Primary secondary amine
PT	Pleuromutilines
QDs	Quantum Dots
QN	Quinolones
QNX	Quinoxaline
QuEChERS	Quick, easy, cheap, effective, rugged, and safe
Q-Orbitrap	Quadrupole-Orbitrap mass spectrometer
QqQ	Triple quadrupole mass detector
QToF	Quadrupole Time-of-Flight mass spectrometer
RASFF	Rapid Alert System for Food and Feed
AS	Sulfonamides (including Dapsone)
SBSE	Stir-bar sorptive extraction
SDZ	Sulfadiazine
SL	Screening limit
SLE	Solid–Liquid Extraction
SMX	Sulfamethoxazole
SMZ	Sulfamethazine
SOSLE	Salting-out supported liquid extraction
SPE	Solid phase extraction
SPME	Solid-phase microextraction
SPR	Surface Plasmon Resonance
SQX	Sulfaquinoxaline
STC	Screening Target Concentration
TAP	Thiamphenicol
TC	Tetracyclines
TCA	Trichloroacetic acid
TFA	Trifluoroacetic acid
TQ	Tranquilizers
TT	Anti-thyroids
ToF	Time of Flight
UHPLC-QqQ	UHPLC coupled to triple quadrupole mass spectrometry
UHPLC	Ultra-High-Performance Liquid Chromatography
UAE	Ultrasonic-assisted extraction
UCNPs	Upward Converting Nanoparticles
UHPLC–MS/MS	Ultra-High-Performance Liquid Chromatography tandem Mass Spectrometry
UHPLC-Q/ToF	UHPLC coupled to Time of Flight mass spectrometry
VMPs	Veterinary Medicinal Products
